# Identification of a novel anoikis signalling pathway using the fungal virulence factor gliotoxin

**DOI:** 10.1038/s41467-018-05850-w

**Published:** 2018-08-30

**Authors:** Florian Haun, Simon Neumann, Lukas Peintner, Katrin Wieland, Jüri Habicht, Carsten Schwan, Kristine Østevold, Maria Magdalena Koczorowska, Martin Biniossek, Matthias Kist, Hauke Busch, Melanie Boerries, Roger J. Davis, Ulrich Maurer, Oliver Schilling, Klaus Aktories, Christoph Borner

**Affiliations:** 1 Institute of Molecular Medicine and Cell Research, Faculty of Medicine, Albert Ludwigs University Freiburg, Stefan Meier Strasse 17, 79104 Freiburg, Germany; 2 Faculty of Biology, Albert Ludwigs University Freiburg, Schänzlestrasse 1, 79104 Freiburg, Germany; 3 Spemann Graduate School of Biology and Medicine (SGBM), Albert Ludwigs University Freiburg, Albertstrasse 19a, 79104 Freiburg, Germany; 4 Institute of Immunology, University Hospital Heidelberg, Im Neuenheimer Feld 305, 69120 Heidelberg, Germany; 5 Institute of Experimental and Clinical Pharmacology and Toxicology, Albert Ludwigs University Freiburg, Albertstrasse 25, 79102 Freiburg, Germany; 6 German Cancer Consortium (DKTK), German Cancer Research Center (DKFZ), INF 280, 69120 Heidelberg, Germany; 7 Howard Hughes Medical Institute & Program in Molecular Medicine, University of Massachusetts Medical School, Worcester, MA 01605 USA; 8 BIOSS Centre for Biological Signalling Studies, Schänzlestrasse 14, 79104 Freiburg, Germany; 9 Present Address: Institute of Experimental Dermatology and Institute of Cardiogenetics, University of Lübeck, Ratzeburger Allee 160, 23538 Lübeck, Germany

## Abstract

Anoikis is a form of apoptosis induced by cell detachment. Integrin inactivation plays a major role in the process but the exact signalling pathway is ill-defined. Here we identify an anoikis pathway using gliotoxin (GT), a virulence factor of the fungus *Aspergillus fumigatus*, which causes invasive aspergillosis in humans. GT prevents integrin binding to RGD-containing extracellular matrix components by covalently modifying cysteines in the binding pocket. As a consequence, focal adhesion kinase (FAK) is inhibited resulting in dephosphorylation of p190RhoGAP, allowing activation of RhoA. Sequential activation of ROCK, MKK4/MKK7 and JNK then triggers pro-apoptotic phosphorylation of Bim. Cells in suspension or lacking integrin surface expression are insensitive to GT but are sensitised to ROCK-MKK4/MKK7-JNK-dependent anoikis upon attachment to fibronectin or integrin upregulation. The same signalling pathway is triggered by FAK inhibition or inhibiting integrin αV/β3 with Cilengitide. Thus, GT can target integrins to induce anoikis on lung epithelial cells.

## Abstract (web summary)

Gliotoxin (GT), produced by the pulmonary pathogen *A. fumigatus*, induces detachment-induced apoptosis (anoikis) of lung epithelial cells and likely promotes invasion. Here, the authors show that GT covalently modifies integrins at the RGD binding site followed by activation of RhoA-ROCK-MKK4/7-JNK signalling leading to Bim-mediated anoikis.

## Introduction

Detachment-induced apoptosis or anoikis is a crucial process to eliminate aberrant cells in the outer layer of epithelia^1^. Lack of anoikis is a hallmark of cancer progression as cells that continue to survive in suspension are prone to metastasise^1^. Originally described by Frisch et al.^2^, anoikis is typically induced by detaching cells with trypsin and preventing their re-attachment to polyHEMA-coated plates. However, this system is artificial as trypsin inappropriately modifies adhesion molecules leading to the activation of signalling pathways that may not reflect physiological ways of anoikis.

Cells attach to the extracellular matrix via integrins. Integrins consist of transmembrane α and β chains, which form at their extracellular N termini an interaction interface with the Arg-Gly-Asp (RGD) motif of matrix components such as fibronectin or vitronectin^3^. On the intracellular side integrins recruit components that link adhesion signals to cell survival, cell cycle control and cytoskeletal rearrangement^1,4,5^. Key players are focal adhesion kinase (FAK)^6^, integrin-linked kinase (ILK)^7^ and Src tyrosine kinase^8^. FAK is autophosphorylated at Y397 upon integrin activation^9^ and subsequently phosphorylates adapter molecules such as paxillin, vinculin and Rho-, Rac- and Cdc42 GTPase-modulating proteins to regulate the actin cytoskeleton^1,6,10^. FAK and ILK further activate PI3K/AKT, ERK/MAPK and JNK signalling pathways^6,7,11–13^.

Apoptosis can be induced by extrinsic death receptor signalling where activated receptors of the tumour necrosis facto superfamily recruit and activate caspase-8 via FADD leading to the cleavage and activation of effector caspase-3^14^. Alternatively, so called BH3-only proteins of the Bcl-2 family sense apoptotic signals and convey them to Bax/Bak-mediated mitochondrial outer membrane permeabilization^15^. The subsequent release of cytochrome c induces the formation of the Apaf-1/caspase-9 apoptosome, which results in caspase-3 activation. Although evidence was initially presented that death receptor signalling via FasL^16^ and/or FADD^17^ plays a role in anoikis induction, this could not be confirmed in other studies^18–20^. By contrast, several cellular processes seem to block mitochondrial apoptosis signalling in adherent cells^19^. Activation of AKT, elicited by integrin engagement, leads to the phosphorylation of the BH3-only proteins Bad^21^ and Bim^22^, which are sequestered and inactivated by 14-3-3 proteins. AKT also inhibits Forkhead transcription factors (FOXOs)^23^, which are responsible for the transcriptional upregulation of the BH3-only proteins Bim, Puma and Bmf^24^. Moreover, both ERK-^25^ and PI3K/AKT-mediated phosphorylation^24^ of Bim lead to its proteasomal degradation. However, it has been unclear if activation of these BH3-only proteins during anoikis is indeed linked to AKT and/or ERK inhibition.

The mould *Aspergillus fumigatus* causes a severe pulmonary disease termed invasive aspergillosis^26^. Under healthy conditions, airborne conidia released by *A. fumigatus* are successfully eliminated from the pulmonary cavities by alveolar macrophages, neutrophils and leucocytes^27^. In immunosuppressed patients, however, *A. fumigatus* germinates, invades the lung and causes severe and often lethal systemic infections^26,27^. The breakage of the epithelial barrier is the most likely cause for the invasive property of *A. fumigatus*. Accumulating evidence suggests a crucial role of the major virulence factor gliotoxin (GT) in this process because fungi lacking GT production are much less virulent than wild-type (WT) strains^28^. We previously showed that GT induces a rapid detachment of human lung epithelial cells and mouse fibroblasts before they undergo caspase-dependent apoptosis^29,30^. GT-induced apoptosis requires a JNK-mediated triple phosphorylation of Bim at S100/T112/S114, which increases the pro-apoptotic activity of Bim^30^. Moreover, both in vitro and in vivo, GT-induced cytotoxicity depended on Bak^29^ indicating that epithelial barrier breakage and lung invasion after *A. fumigatus* infection may be due to GT-mediated anoikis.

Here we use GT to delineate for the first time an entire anoikis signalling pathway in human lung epithelial cells that leads to the direct activation of the pro-apoptotic family member Bim. GT covalently modifies the RGD-binding domain of integrin α and β chains, leading to rapid cell detachment followed by FAK inactivation and subsequent activation of a RhoA-ROCK-MKK4/MKK7-dependent signalling pathway, which activates JNK- and Bim-mediated apoptosis.

## Results

### GT employs MKK4 and MKK7 to activate JNK-dependent apoptosis

**Fig. 1:**
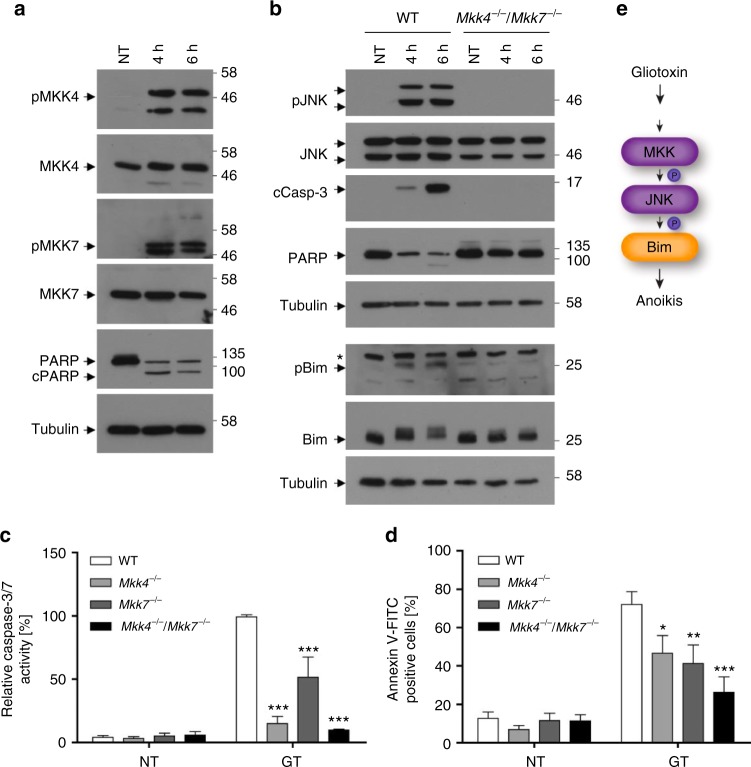
MKK4 and MKK7 are required for GT-induced anoikis. **a** Western blot analysis of total extracts of human bronchial epithelial cells (BEAS-2B) showing increased phosphorylation of MKK4 (Ser257/Thr261) (pMKK4) and MKK7 (Ser271/Thr275) (pMKK7) as well as PARP cleavage (PARP/cPARP) after GT treatment for 4 and 6 h. **b** Western blot analysis showing increased phosphorylation of JNK (T183/Y185) (pJNK) and Bim (T112/S114) (pBim) and enhanced processing of caspase-3 and PARP in total extracts of WT MEFs treated with GT for 4 and 6 h. None of these changes were seen in the extracts of non-treated (NT) cells or MEFs deficient for both *Mkk4* and *Mkk7* (*Mkk4*^*−/−*^*/Mkk7*^−/−^). **c**, **d** MEFs deficient for either *Mkk4* (*Mkk4*^*−/−*^), *Mkk7* (*Mkk7*^*−/−*^) or both *Mkk4/Mkk7* (*Mkk4*^*−/−*^*/Mkk7*^*−/−*^) exerted reduced caspase-3/7 activity (**c**) and annexin V-FITC staining (**d**) and as compared to wild-type (WT) cells when treated with GT for 6 h. **e** Schematic representation of how GT activates MKK4/MKK7 (MKK) and triggers JNK/Bim-mediated anoikis. Tubulin was used as loading control in **a** and **b**. Graphs in **c** and **d** show the means of at least three independent experiments ± s.e.m.; *p*-values: *0.05–0.01, **0.01–0.001, ***<0.001; two-way ANOVA, post hoc: Bonferroni compared to WT

We previously reported that JNK is required for GT-induced apoptosis^30^. We therefore sought to identify the kinase(s) responsible for JNK activation. Possible candidates were the mitogen-activated protein kinases MKK4 and MKK7. Indeed, after 4–6 h of GT treatment of human bronchial epithelial cells (BEAS-2B) both MKK4 and MKK7 were phosphorylated in their activation loops (S257/T261 and S271/T275, respectively) as detected by phosphospecific antibodies (Fig. 1a). This coincided with the cleavage of the caspase-3 substrate PARP.

To determine if MKK4 and/or MKK7 were required for GT-induced JNK activation and apoptosis, we analysed WT, *Mkk4*^−/−^, *Mkk7*^−/−^ and *Mkk4*^*−/−*^*/Mkk7*^*−/−*^ mouse embryonic fibroblasts (MEFs). While WT MEFs exhibited a marked increase in caspase-3/7 activity (Fig. 1c) and cell death (Fig. 1d) after 6 h of GT treatment, this was less the case for *Mkk4*^*−/−*^ and *Mkk7*^*−/−*^ cells. MEFs deficient for both *Mkk4* and *Mkk7* showed the highest degree of protection against GT-induced caspase-3 activation and cell death (Fig. 1c, d). Western blot analysis confirmed that MKK4 and MKK7 were required for phosphorylation of JNK in its activation loop (Thr183/Tyr185), JNK-mediated triple phosphorylation of Bim (pBim) and caspase-3 processing to the active p17 form (cCasp-3) since all these effects were completely ablated in GT-treated *Mkk4*^*−/−*^*/Mkk7*^*−/−*^ MEFs (Fig. 1b). Thus, both MKK4 and MKK7 link GT to JNK activation along the anoikis signalling pathway (Fig. 1e).

### GT triggers a Rho-dependent phosphorylation cascade

Since GT causes rapid cell detachment associated with cytoskeletal changes (Supplementary Fig. 1), we looked for an upstream MKK4/MKK7 activator, which is linked to these events. Recent evidence indicated that Rho-related small GTPases such as RhoA, Rac1 and Cdc42 do not only control actin remodelling but also the activity of the JNK cascade^31^. This prompted us to investigate if the Rho-associated protein kinase (ROCK) was involved in GT-induced MKK4/MKK7 activation and detachment-induced cell death.

**Fig. 2:**
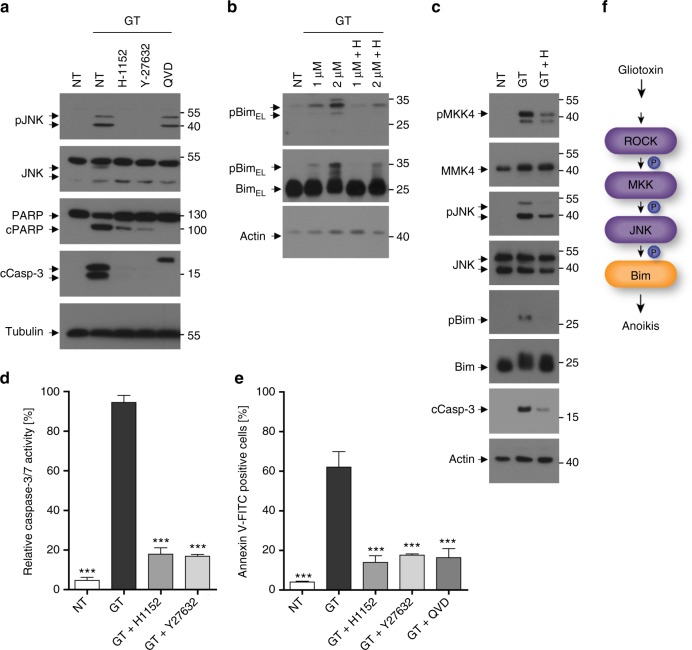
ROCK is required for GT-induced anoikis. **a**, **b** Western blot analysis showing that the pre-treatment of BEAS-2B cells with the ROCK inhibitors H-1152 (1 µM) or Y-27632 (1 µM) abrogated GT-induced JNK phosphorylation and caspase-3 and PARP processing (**a**) as well as Bim phosphorylation (**b**). Treatment with 25 µM QVD prevented caspase-3 and PARP processing but not JNK phosphorylation. **c** Western blot analysis showing that the pre-treatment of MEFs with the ROCK inhibitor H-1152 diminished GT-induced MKK4 and JNK phosphorylation, Bim phosphorylation and caspase-3 processing. **d**, **e** Both ROCK inhibitors prevented GT-induced caspase-3/7 activity (**d**) and apoptosis (as measured by annexin V-FITC staining) (**e**) in MEFs to the same extent as the general caspase inhibitor QVD (25 µM). **f** Schematic representation of how GT activates ROCK and triggers a MKK4/MKK7-JNK-Bim-mediated anoikis signalling pathway. Tubulin (**a**) and actin (**b**, **c**) were used as loading controls. Graphs in **d** and **e** show the means of at least three independent experiments ± s.e.m.; *p*-values: ***<0.001, one-way ANOVA, post hoc: Bonferroni compared to GT

For that purpose, we treated BEAS-2B cells with two pharmacological ROCK inhibitors, H-1152 and Y-27632, before applying GT for 6 h. Both inhibitors completely abolished GT-induced JNK phosphorylation and caspase-3 and PARP processing (Fig. 2a) as well as Bim phosphorylation at T112/S114 (Fig. 2b). An in vitro JNK activity assay showed that GT-induced c-Jun phosphorylation was ablated after H-1152 treatment (Supplementary Fig. 2E and 2F). Importantly, the general caspase inhibitor QVD did not affect GT-induced JNK phosphorylation but expectedly blocked caspase-3 activation (Fig. 2a).

Similar results were obtained in MEFs. H-1152 diminished MKK4 and JNK activation, Bim phosphorylation and caspase-3 processing after GT treatment (Fig. 2c). Consequently, both ROCK inhibitors abrogated caspase-3/7 activity (Fig. 2d) and apoptosis (Fig. 2e) to the same extent as the caspase inhibitor QVD indicating that GT-induced caspase-3 activation was caused by increased ROCK activity.

To ensure that the observed effects of H-1152 and Y-27632 were due to ROCK inhibition, we effectively knocked down ROCK1 by short hairpin (shRNA) in BEAS-2B cells (Supplementary Fig. 2A). Depletion of ROCK1 prevented GT-induced JNK phosphorylation and caspase-3 processing (Supplementary Fig. 2D) as well as caspase-3/7 activity (Supplementary Fig. 2B) and apoptosis (Supplementary Fig. 2C). Thus, ROCK links GT to MKK4/MKK7 and JNK activation along the anoikis signalling pathway (Fig. 2f).

**Fig. 3:**
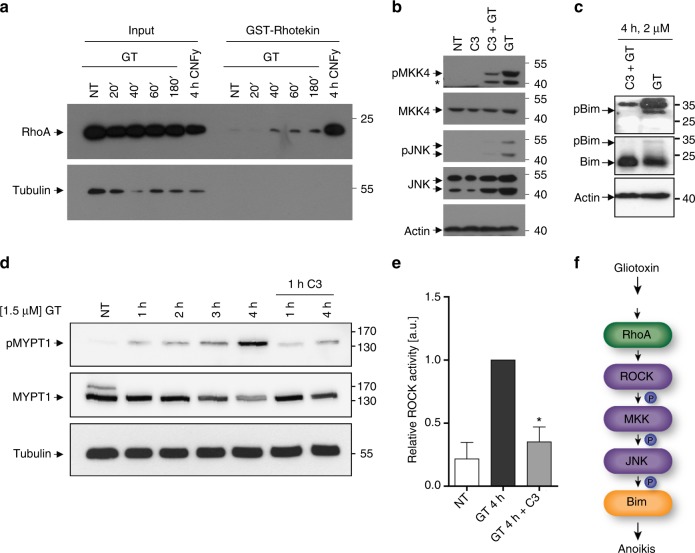
GT triggers RhoA and subsequent ROCK activation. **a** Anti-RhoA western blot analysis showing the pulldown of active RhoA-GTP with GST-Rhotekin beads from total extracts of BEAS-2B cells either treated with 1 µM GT for 20, 40, 60 and 180 min or with the bacterial toxin CNFy for 4 h (positive control). The left side of the western blots shows equal RhoA input for each pulldown. **b** Western blot analysis of total extracts of BEAS-2B cells either untreated (NT) or treated with 1 µM GT in the absence or presence of the RhoA-inhibiting C3 toxin for 6 h showing a diminishment of GT-induced MKK4 and JNK phosphorylation by the C3 toxin. **c** Western blot analysis of total extracts of BEAS-2B cells treated with 2 µM GT in the absence or presence of the C3 toxin for 4 h showing that the toxin diminishes GT-induced Bim phosphorylation at T112/S114 (pBim). **d** Anti-MYPT1 and pMYPT1 western blot analysis of total extracts of BEAS-2B cells either untreated (NT) or treated with 1.5 µM GT in the absence or presence of the C3 toxin for 1–4 h showing that the toxin ablated the GT-induced phosphorylation of the ROCK substrate MYPT1. **e** Lumi Imager FusionSL Vilber Lourmat quantification of the chemiluminescent immunoblot bands shown in **d**. **f** Schematic representation of how GT activates RhoA and subsequently triggers a ROCK-MKK4/MKK7-JNK-Bim-dependent anoikis signalling pathway. Actin (**b**, **c**) and tubulin (**a**, **d**) were used as loading controls. Graph in **e** shows the means of at least three independent experiments ± s.e.m.; *p*-values: *0.05–0.01, one-way ANOVA, post hoc: Bonferroni compared to GT

We next examined the role of ROCK-activating Rho GTPases in GT-induced anoikis signalling. For that purpose, we took advantage of Rhotekin, a known binding partner and substrate of RhoA and RhoC^32^. We performed GST-Rhotekin pulldowns of untreated and GT-treated BEAS-2B cell extracts and examined them for the abundance of active RhoA. As a positive control, we used the bacterial toxin CNFy, which inhibits the GTPase activity of Rho proteins, thereby keeping them in a permanent GTP-bound active state (Fig. 3a)^33^. As shown in Fig. 3a, the amount of active RhoA-GTP in the GST-Rhotekin pulldowns started to increase after 40 min of GT treatment, a time that conincided with GT-induced cell detachment (Supplementary Fig. 1). To confirm that increased Rho activity was crucial for GT-induced anoikis signalling, we treated BEAS-2B cells with the bacterial toxin C3, which inhibits Rho activity by ADP ribosylation^34^. Rho inhibition by C3 reduced GT-induced phosphorylation of MKK4 and JNK (Fig. 3b) as well as the phosphorylation of Bim at its T112/S114 JNK phosphorylation sites (Fig. 3c).

Finally, we wanted to know if the C3 toxin had any inhibitory effect on GT-induced ROCK activation. We therefore monitored the phosphorylation of a major substrate of ROCK, myosin-binding subunit of myosin phosphatase (MYPT1)^31^ by western blot analysis. As shown in Fig. 3d, e, while the phosphorylation of MYPT1 gradually increased after GT treatment, this was not the case when BEAS-2B cells were pretreated with the C3 toxin. Thus, GT triggers Rho activation (particularly RhoA) to stimulate ROCK-MKK4/MKK7-JNK-Bim-dependent anoikis signalling (Fig. 3f).

### GT disrupts the focal adhesion complex

**Fig. 4:**
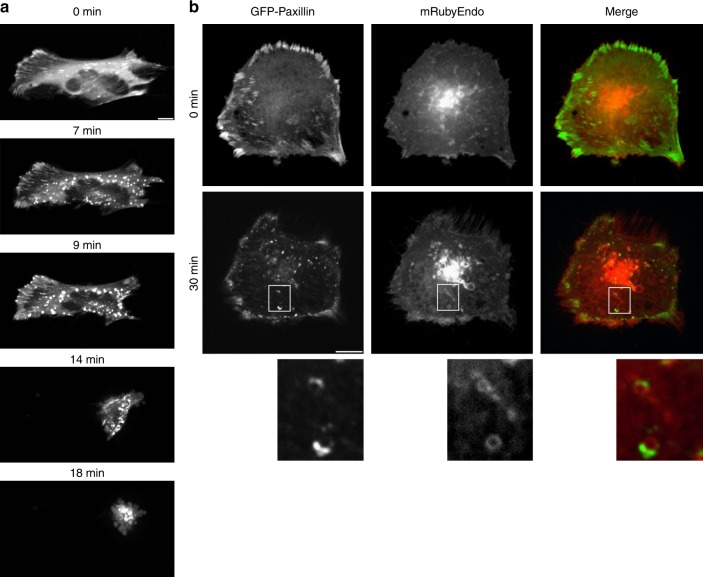
Paxillin translocates from focal adhesions to early endosomes. **a** GT-induced cell detachment was studied by confocal video time-lapse microscopy. In response to 1 µM GT, GFP-paxillin stably transfected into BEAS-2B cells translocates from focal adhesions at the plasma membrane (0 min) to vesicles (7 min), which move into the cell (7–18 min) (also see Supplementary Movie 1). This is followed by cell rounding and detachment (14–18 min). **b** Co-transfection of BEAS-2B cells with GFP-paxillin and mRuby-Endo-14 to visualise endosomes. Paxillin partially localised at endosomal membranes (see insets at higher digital magnification). Scale bar = 10 µm

Adherent cells treated with GT rapidly detach before they die (Supplementary Fig. 1)^30^. This suggests that disruption of focal adhesions might be an early event of GT action. Paxillin is a scaffold protein at the cytoplasmic side of focal adhesions responsible for recruiting FAK, a crucial mediator of integrin signalling^35^. We used green fluorescent protein (GFP)-labelled paxillin to monitor its subcellular localisation before and after GT treatment by confocal time-lapse microscopy. While in adherent healthy BEAS-2B cells, GFP-paxillin was present in focal adhesions as well as in the cytosol (Fig. 4a), it mainly localised to vesicular structures after GT treatment before the cell rounded up and died (Fig. 4a and Supplementary Movie 1). Co-transfection with the endosomal marker mRuby-Endo-14 revealed that GFP-paxillin resided on endosomal membranes after GT treatment (Fig. 4b).

Since GT changed the structure/composition of focal adhesions, we sought to study the role of FAK in GT-induced apoptosis. FAK is an interesting downstream target of GT because it is known to regulate Rho GTPases during stress fibre formation and focal adhesion turnover^36^. Moreover, it is known to phosphorylate and activate p190RhoGAP, which negatively regulates Rho activity in adherent cells^36^. We therefore investigated (i) if FAK activity was regulated by GT and (ii) if this affected the activity state of RhoA and therefore the RhoA-ROCK-MKK4/MKK7-JNK-mediated anoikis signalling.

**Fig. 5:**
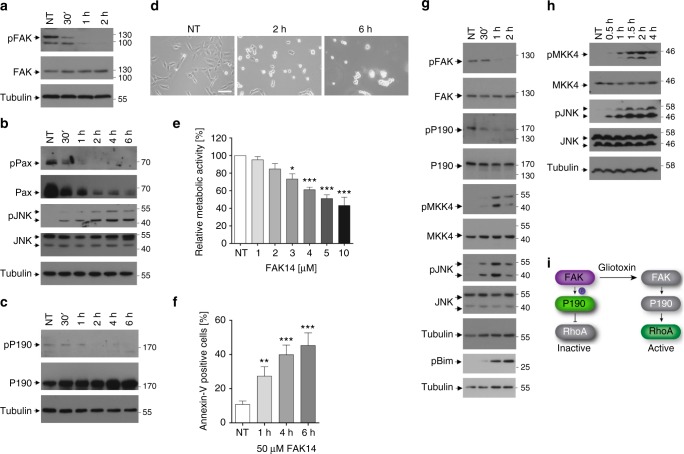
FAK and p190RhoGAP are inactivated by GT. **a** Western blot analysis of total extracts of BEAS-2B cells either untreated (NT) or treated with 1 µM GT for the indicated time periods showing that the activating phosphorylation of FAK at Y397 (pFAK) was lost within 1 h. **b** Paxillin (pPax) was dephosphorylated and degraded and JNK was phosphorylated/activated (pJNK) with the same kinetics as FAK inactivation. **c** Concomitantly, p190RhoGAP was also dephosphorylated at Y1105. **d** Treating BEAS-2B cells with 10 µM of the specific FAK inhibitor FAK14 caused rapid cell detachment within 6 h. Scale bar = 100 µm. **e**, **f** Cell survival of BEAS-2B cells measured as relative metabolic activity using the XTT assay gradually decreased with increasing doses of FAK14 (0–10 µM) (**e**). Concomitantly, 50 µM FAK14 triggered cell death of BEAS-2B cells (as measured by annexin V-FITC staining) with similar time kinetics as GT (**f**). **g** Western blot analysis of total extracts of BEAS-2B cells either non-treated (NT) or treated with 50 µM of FAK14 (**g**) or 10 µM of FAK14 (**h**) for 30 min to 4 h. The inhibitor caused FAK and p190RhoGAP dephosphorylation and MKK4, JNK and Bim phosphorylations. Tubulin was used as loading control in **a**–**c**, **g** and **h**. **i** FAK is active in adherent epithelial cells causing the activation of p190RhoGAP by its phosphorylation at Y1105. This inactivates RhoA. In response to GT, FAK and p190RhoGAP get inactivated, therefore allowing RhoA activation. Graphs in **e** and **f** show the means of at least three independent experiments ± s.e.m.; *p*-values: *0.05–0.01, **0.01–0.001, ***<0.001, one-way ANOVA, post hoc: Bonferroni compared to NT

Within 30 min of GT treatment of BEAS-2B cells, the activating phosphorylation of FAK at Y397 was lost (Fig. 5a). Simultaneously, the phosphorylations of its substrates p190RhoGAP (Fig. 5c) and paxillin (Fig. 5b) diminished and paxillin was degraded (Fig. 5b). Dephosphorylation of p190RhoGAP results in a lower GAP activity towards Rho proteins, therefore favouring their active GTP-bound state^36^. Indeed, we observed a higher level of active RhoA (Fig. 3a) and phosphorylation of its downstream target JNK (Fig. 5b) at the time of FAK inactivation (30–60 min). Thus, GT-induced RhoA and JNK activation involves FAK and p190RhoGAP dephoshorylation/inactivation (Fig. 5i).

### Inhibition of FAK mimics GT-induced anoikis signalling

If inactivation of FAK by GT triggered apoptosis via Rho-ROCK-MKK4/7-JNK activation, pharmacological inhibition of FAK should activate the same signalling pathway. We therefore used the FAK inhibitor FAK14, which is highly specific for FAK because it blocks recruitment of its downstream target Src at Y397^37^. As shown for GT, FAK14 induced cell detachment (Fig. 5d) and apoptotic cell death of BEAS-2B cells within 6 h at low (3–10 μM) and high (50 μM) doses (Fig. 5e, f). In addition, both doses of FAK14 caused the rapid dephosphorylation of FAK at Y397, dephosphorylation and hence inactivation of p190RhoGAP, phosphorylation of MKK4 and JNK (Fig. 5g, h) and phosphorylation of Bim at T112/S114 (Fig. 5g). Hence FAK inhibition stimulated the same anoikis signalling pathway as GT (Fig. 5i).

To confirm that inactivation of FAK was crucial for GT-induced JNK activation and caspase-mediated apoptosis, we overexpressed WT FAK or activated mutant forms of FAK (myrFAK, superFAK (FAK K578E/K581E) and myr-superFAK)^38,39^ in BEAS-2B cells by lentiviral transduction. All variants of FAK were overexpressed to similar levels, were phosphorylated at Y397 and triggered enhanced phosphorylation of their substrate p130Cas^38^ confirming their high kinase activities (Supplementary Fig. 3A and 3C). As a consequence, GT-induced anoikis signalling, i.e., JNK and Bim phosphorylation and caspase-3 processing and activation were delayed (Supplementary Fig. 3A, 3B, 3D and 3E). However, anoikis signalling was not fully blocked most likely because GT could still dephosphorylate and inactivate myrFAK, superFAK and myr-superFAK (Supplementary Fig. 3A and 3C). This finding is consistent with previous observations that the downstream signalling of superFAK still depends on integrin-mediated adhesion^39^.

ROCK has been reported to regulate focal adhesions via an inside-out cytoskeletal signalling^40^. We therefore examined if the ROCK inhibitor H-1152 or the RhoA inhibitor toxin C3 had any effect on FAK phosphorylation and cell detachment. The inhibitors themselves did not detach BEAS-2B cells (Supplementary Fig. 1) and H-1152 induced a minor FAK dephosphorylation/inactivation without affecting JNK phosphorylation (Supplementary Fig. 2G) as reported before^40^. In the presence of GT, both H-1152 and C3 slightly delayed cell detachment (Supplementary Fig. 1). This did however not affect focal adhesions since FAK was similarly dephosphorylated by GT irrespective of the presence of absence of H-1152 (Supplementary Fig. 2G). Only JNK and caspase-3 failed to be activated in the presence of the ROCK inhibitor (Supplementary Fig. 2G) confirming that ROCK is a downstream mediator of GT-induced apoptosis and not a major inside-out signalling regulator of cell detachment in this system.

### GT can directly target integrins at the RGD binding site

Since GT caused cell detachment and inactivation of the focal adhesion complex, it may directly target integrins. GT contains an intramolecular disulphide bond essential for its cytotoxic activity^28^. It may therefore covalently modify cysteine residues in integrin α and β chains, which are critical for integrin activation and/or their binding to extracellular matrix components^41,42^. For that purpose, we incubated recombinant human integrin αVβ3 with GT and determined peptides with possible GT-cysteine adducts by mass spectrometry (MS) analysis. Two cysteines were found to be modified by GT, Cys158 in the seven blade β-propeller domain of αV and Cys258 in the ligand-binding β-I domain of β3 integrins (Supplementary Fig. 4A). Both cysteines are highly conserved among the α and β chains of various integrins (Supplementary Fig. 4B) and form intracellular disulphide bridges (Cys158 with Cys138 in αV and Cys258 with Cys299 in β3)^43,44^ that determine efficient binding of integrins to the RGD motif in fibronectin and vitronectin^41,42^. To provide further evidence that GT modified integrins at cysteines in the RGD binding site, we treated BEAS-2B cells with GT for 30 min and subjected a total cellular extract to anti-GT immunoprecitations (IPs) followed by anti-integrin αV or β1 western blot analysis. As shown in Supplementary Fig. 5A and 5B, both integrin chains were specifically detected in anti-GT as compared to control IgG1 IPs. This was however not the case when the cells were pretreated with an RGD peptide before GT addition, or the extract was incubated with dithiothreitol (DTT) and iodoacetamide before anti-GT IP (Supplementary Fig. 5A and 5B). Interestingly, GT seemed to also interact with E-cadherin (Supplementary Fig. 5C) but not with the epidermal growth factor receptor (EGFR) (Supplementary Fig. 5D).

**Fig. 6:**
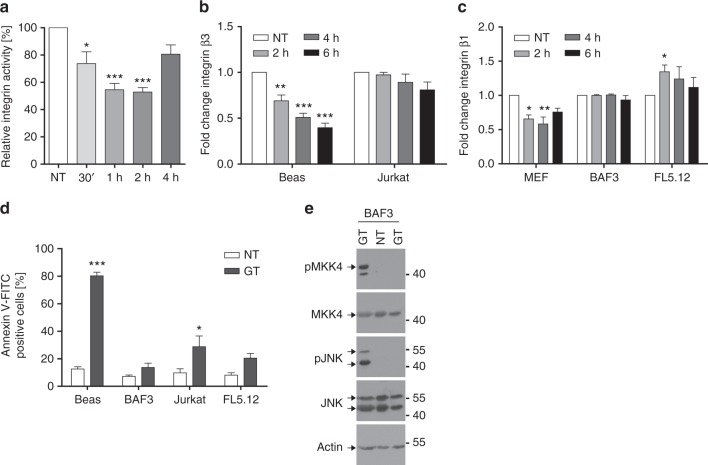
GT diminishes RGD binding and surface expression of integrins. **a** The binding capacity of integrins to extracellular matrix components was measured on untreated (NT) and GT-treated BEAS-2B cells by FACS analysis of RGD-FAM peptide staining. **b**, **c** Adherent human lung epithelial cells (BEAS-2B) or MEFs showed reduced surface expression of integrin β3 (**b**) or integrin β1 (**c**), respectively, after 2–6 h of GT treatment as evidenced by FACS analysis of anti-integrin-stained cells. Human Jurkat (**b**) or mouse BAF or FL5.12 suspension cells (**c**) did not show such a reduction although they expressed integrins on their surface (Supplementary Fig. 7A and 7B), most likely in an inactive form (Supplementary Fig. 7C). **d** While BEAS-2B cells underwent apoptosis (as determined by annexin V-FITC FACS analysis) after 1 µM GT treatment for 6 h, this was not the case for BAF3, Jurkat or FL5.12 suspension cells. **e** Western blot analysis of total extracts of BEAS-2B cells (first lane) or BAF3 cells either untreated (NT) or treated with 1 µM GT for 6 h showing that GT did not cause phosphorylation/activation of MKK4 and JNK in BAF3 but does so in BEAS-2B cells. Actin was used a loading control. Graphs in **a**–**d** show the means of at least three independent experiments ± s.e.m.; *p*-values: *0.05–0.01, **0.01–0.001, ***<0.001, two-way ANOVA, post hoc: Bonferroni compared to NT

To confirm that integrin binding to extracellular matrix components is indeed perturbed by GT, we determined integrin-binding capacity of untreated and GT-treated BEAS-2B cells by fluorescence-activated cell sorting (FACS) analysis using a fluorescently labelled RGD peptide (Cyclo-RGD-5-FAM). As shown in Fig. 6a, integrin binding to RGD-FAM rapidly diminished 30 min to 2 h after GT treatment. This kinetic coincided with the time course of FAK and p190RhoGAP inactivation (Fig. 5a, c), paxillin translocation (Fig. 4a) and cell detachment (Supplementary Fig. 1). By confocal immunofluorescence analysis using antibodies against pFAK and the endosomal protein EEA1 as well as GFP-paxillin overexpression we further showed that phosphorylated FAK (pFAK) colocalized with paxillin in focal adhesions in untreated cells (Supplementary Fig. 6B). In response to GT paxillin is endocytosed (Supplementary Fig. 6B and Fig. 4a) but rarely any pFAK is found on endosomes (Supplementary Fig. 6A). This confirms that after GT treatment FAK gets rapidly dephosphorylated in the focal adhesion plaques at the plasma membrane before it is taken up into the cell together with paxillin.

Integrins are also endocytosed after cell detachment and recycle back to the plasma membrane^45^. We therefore monitored the fate of integrin β3 surface expression on BEAS-2B cells (Fig. 6b) and integrin β1 surface expression on MEFs (Fig. 6c) after GT treatment by FACS analysis using β chain-specific antibodies. Both integrin chains were removed from the cell surface (Fig. 6b, c). This however occurred only after 2 h of GT treatment suggesting that the primary action of GT was to inactivate RGD binding (Fig. 6a) before integrins were endocytosed.

### GT-induced anoikis does not occur in suspension cells

**Fig. 7:**
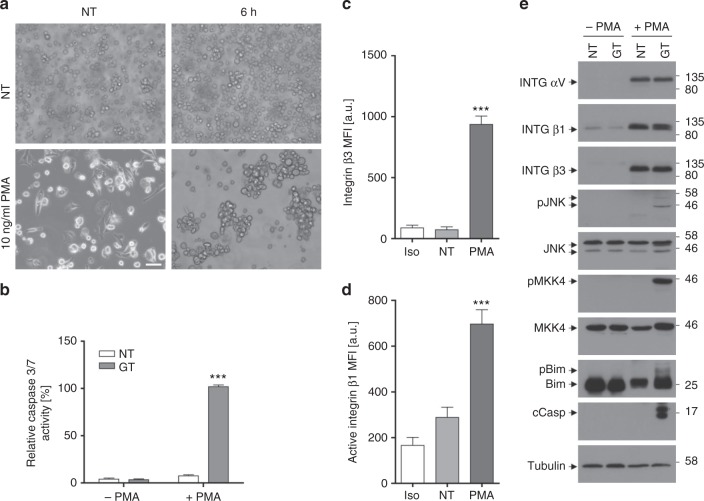
Induction of integrins by PMA confers sensitivity to anoikis. **a** Phase contrast microscopy analysis of K562 cells treated with 10 ng/ml PMA for 96 h before adding 1 μM GT for 6 h. While untreated (NT) cells grow in suspension, PMA-treated cells adhere to the dish and become sensitive to GT-induced detachment. Scale bar = 100 µm. **b** GT induced caspase-3/-7 activity (taken as 100%) in PMA-treated adherent K562 cells (+PMA) but not in undifferentiated suspension cells (−PMA). **c**, **d** FACS analysis of K562 cells showing that PMA increased the surface expression of integrin β3 (**c**) and an active form of integrin β1 (using the anti-active integrin β1 12G10 Alexa Fluor^®^ 488) (**d**). Iso: respective Ig control-FITC antibody. **e** Western blot analysis of total extracts of undifferentiated (−PMA) and differentiated (+PMA) K562 cells either untreated (NT) or treated with 1 µM GT for 6 h showing that PMA induced the expression of integrin (INTG) αV, β1 and β3. GT triggered the phosphorylation of MKK4, JNK and Bim (as evidence by a gel shift) as well as caspase-3 processing, but only in PMA-treated cells. Tubulin was used a loading control. Graphs in **b**–**d** show the means of at least three independent experiments ± s.e.m.; *p*-value: ***<0.001, two-way ANOVA, post hoc: Bonferroni compared to non-treated (NT)

Suspension cells do usually not express active integrins on their surface and hence do not form mature focal adhesions. Therefore, these cells should not be killed by GT if the toxin specifically targets focal adhesions in adherent cells. Indeed, while BEAS-2B cells effectively underwent apoptosis in response to GT, three different human and mouse suspension cell lines, BAF3, Jurkat and FL5.12, were insensitive to GT-induced apoptosis (Fig. 6d). Consistent with this finding, neither MKK4 nor JNK was phosphorylated/activated (Fig. 6e) and caspase-3 was not processed (Supplementary Fig. 8D). All cell lines expressed integrins β1 and β3 on their surface (Fig. 6a–c, Supplementary Fig. 7A and 7B). On BEAS-2B cells, they were in an active state as evidenced by RGD-FAM (Fig. 6a) and anti-active integrin β1 staining (Supplementary Fig. 7C). This was however not the case on Jurkat cells where integrin β1 was not detected with an anti-active integrin antibody (Supplementary Fig. 7C). Moreover, while GT diminished surface staining of integrins β3 (Fig. 6b) and β1 (Fig. 6c) on BEAS-2B and MEF, respectively, none of the suspension cell lines showed such an effect. To further substantiate the integrin-dependence of GT for its pro-apoptotic action, we made use of K562 cells, which also grow in suspension but express very little integrin β1 and no integrin αV and β3 at all (Fig. 7e). Again, GT could not trigger caspase-3 activity (Fig. 7b) or processing (Fig. 7e) in these cells. However, when the cells were treated with 10 ng/ml phorbol 12-myristate 13-acetate (PMA), they largely upregulated active integrins αV, β1 and β3 on their surface (Fig. 7c–e) and became sensitive to GT-induced cell detachment (Fig. 7a), MKK4, JNK and Bim phosphorylation and caspase-3 processing (Fig. 7e) and activation (Fig. 7b). Similarly, BAF3 suspension cells made adherent by plating them on fibronectin-coated plates overnight displayed activated surface integrins as evidenced by RGD staining (Supplementary Fig. 8B) and acquired sensitivity to GT-induced detachment (Supplementary Fig. 8A), caspase-3 processing (Supplementary Fig. 8D), apoptosis (Supplementary Fig. 8C) and activation of the same MKK4/JNK signalling pathway (Supplementary Fig. 8D) as previously seen in BEAS-2B cells. Hence GT exerts its cytotoxic activity primarily on adherent cells expressing active integrins, which qualifies it as a bona fide anoikis inducer.

### Blocking αV/β3 integrins mimics GT-induced anoikis signalling

If activation of the JNK/Bim-dependent anoikis pathway by GT is a consequence of integrin inactivation, the same pathway should be triggered by integrin inhibitory antibodies or drugs. Cilengitide is an RGD-based compound that primarily blocks integrins αVβ3 and αVβ5 at lower and β1 at higher concentrations^46^. We first confirmed that BEAS-2B cells express αV, β3 and β1 integrins (Supplementary Fig. 7D). When these cells were exposed to 25 μg/ml Cilengitide, they exhibited cell detachment (Supplementary Fig. 7E), FAK dephosphorylation and activation of JNK1/2 with subsequent Bim phosphorylation at T112/S114 (Supplementary Fig 7F and 7G), activation and processing of caspase-3 processing (Supplementary Fig. 7G and 7H) and cell death (Supplementary Fig. 7I) within 6–24 h. However, in contrast to GT, the cells treated with Cilengitide detached in clusters (instead of single cells) (Supplementary Fig. 7E), and FAK dephoshorylation (Supplementary Fig. 7F) and subsequent anoikis signalling were less pronounced and delayed (Supplementary Fig 7G-I). This was also true when Cilengitide was combined with an anti-integrin α5/β1 inhibitory antibody, which was ineffective alone and did not further enhance Cilengitide-induced anoikis (Supplementary Fig. 7I). This might be due to the fact that Cilengitide, even at a concentration where it also affects integrin β1, does not inhibit all forms of integrins. Indeed, Affymetrix Gene Array and RNAseq analysis of BEAS-2B cells revealed the expression of at least 13 integrin genes with ITGB1 (integrin β1), ITGA3 (integrin α3) and ITGAV (integrin αV) being expressed the highest (Supplementary Fig. 4C, 4D and 4E). While GT induces maximal cell detachment and anoikis because it targets cysteines in the RGD binding groove, which are conserved among all integrins, we would need a combination of several inhibitory integrin antibodies to achieve the same effect.

## Discussion

Here we used GT, the major virulence factor of *A. fumigatus*, to identify a novel anoikis signalling pathway. GT qualifies for an anoikis inducer for the following reasons: (i) it induces rapid cell detachment prior to apoptosis induction; (ii) it can directly modify N-terminal cysteines of α and β chains of integrins thereby interfering with their binding to extracellular matrix components; and (iii) it cannot kill suspension cells of the hematopoietic system and does not activate the anoikis signalling pathway in these cells. Only when suspension cells are made adherent, i.e., either plated on fibronectin or induced to express integrins on their surface, they become sensitive to GT-induced anoikis via the same signalling pathway as epithelial cells. Although we provide compelling evidence that GT can directly target integrins by modifying cysteines at the RGD binding interface, we cannot exclude that it also modifies and disrupts binding domains in other adhesion molecules, including inflammatory receptors or cadherins. Indeed, we found that E-cadherin could be co-immunoprecipitated with anti-GT antibodies. This may explain why cells treated with GT detach as single cells while those treated with integrin inhibitory antibodies or compounds detach as cell sheets. Further studies are needed to identify the impact of GT on signalling pathways regulated by cadherins.

Protection from anoikis was suggested to involve activation of ERK/MAPK and PI3K/AKT signalling^11–13^. These pathways should be turned off upon GT action, resulting in dephosphorylation of Bad and Bim and their release from 14-3-3 proteins^21,22^, the transcriptional upregulation of Bim, Puma and Bmf by FOXO activation^23,24^ and the stabilisation of Bim due to lack of proteasomal degradation^25^. However, as we previously reported, neither Bad, Bmf nor Puma was required for GT-induced apoptosis, and the ERK/MAPK and AKT signalling pathways were still transiently activated after GT treatment^30^. Moreover, although Bim was essential for GT-induced cytotoxicity it required JNK-mediated phosphorylation at S100/T112/S114 rather than increased protein stability for effective Bax/Bak activation^30^. This indicated that GT uses a JNK-dependent, but ERK/AKT-independent pathway for anoikis signalling^30^.

Previously, Stupack et al.^47^ reported that unligated integrins or β-integrin tails recruit caspase-8 to the membrane and induce apoptosis distinct from anoikis in death receptor-independent manner. We therefore tested if caspase-8 was activated and required for GT-induced apoptosis. However, while recombinant FasL rapidly activated caspase-8 in BEAS-2B cells, we did not detect any caspase-8 activation after GT treatment for up to 6 h (Supplementary Fig. 9A). Moreover, knocking down caspase-8 expression did not affect the kinetics of GT-induced anoikis but it blocked FasL-induced apoptosis (Supplementary Fig. 9B). Thus, GT uses a JNK-dependent, but ERK/AKT- and caspase-8-independent pathway for anoikis signalling.

JNK/c-Jun-induced transcription in response to lysophosphatidic acid was previously shown to be mediated by RhoA-ROCK-MKK4 signalling^31^. The same pathway was involved in arsenic trioxide-induced apoptosis of chronic myelogenous leukaemia cells^48^. However, in both cases it remained elusive how RhoA was activated. Here we show that RhoA is activated by dephosphorylation and inactivation of p190RhoGAP as a result of GT-induced FAK inhibition. This is consistent with a report showing FAK-induced downmodulation of RhoA activity via p190RhoGAP^36^. The role of Rac or Cdc42 GTPases in mediating GT anoikis could be excluded because they do not activate ROCK^31,40^ and have recently been shown to be inactivated by GT^49^.

ROCK1 and 2 play essential roles in regulating cell morphology, motility and cell fate^31,40,50^. Whether all these effects are mediated through changes in the actin cytoskeleton is still debated^50^. Ethanol, doxorubicin and serum starvation were found to induce caspase-dependent apoptosis via RhoA-ROCK1-mediated myosin light chain phosphorylation and subsequent cytoskeletal rearrangements rather than JNK activation although the link to caspase-3 activation was not provided^50,51^. In other studies ROCK activated JNK but the result was stimulation of cell migration rather than apoptosis^52^. Furthermore, ROCK was shown to affect the membrane blebbing of apoptotic cells^53^. However, in this case ROCK was activated by caspase-3-mediated cleavage, which occurs downstream of cytochrome c release. Although we cannot exclude a cytoskeletal involvement for GT-induced, ROCK-mediated anoikis, our data show that ROCK does not signal back to focal adhesions (FAK) but stimulates the downstream phosphorylation/activation of MKK4 and MKK7 essential for anoikis. The precise mechanism of this activation is not yet understood. It either occurs through intermediate kinases such as MLK or ASK^54,55^ and/or the scaffold proteins hCNK^56^ or JIP-3^57^, which were shown to link ROCK to JNK activation. Once activated MKK4 and MKK7 then phosphorylate different sites in the activation loop of JNK^54^, and we indeed find that both kinases are required for GT-induced JNK activation.

**Fig. 8:**
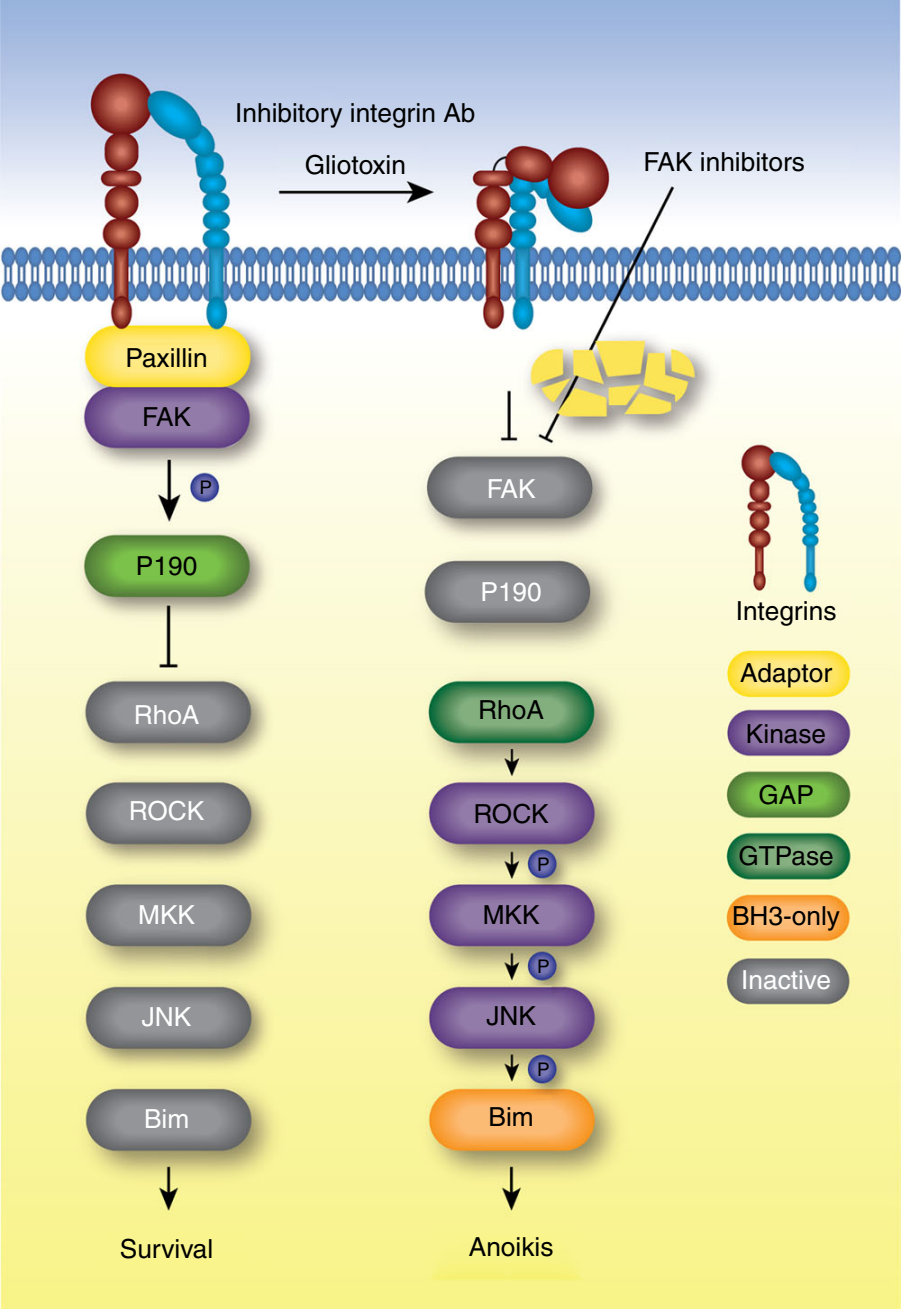
GT-induced anoikis signalling. FAK is recruited to focal adhesions via paxillin and is active in healthy adhesion cells. Active FAK phosphorylates p190RhoGAP. Phosphorylated p190RhoGAP stimulates the GTPase activity of RhoA. RhoA and the downstream anoikis-inducing cascade is therefore inactive (left). GT inactivates integrins, possibly by covalent binding to cysteines in the N-terminal region of α and β chains and triggers the disassembly of focal adhesions. Paxillin is degraded and FAK is inactivated, resulting in inactive p190RhoGAP. RhoA is consequently active and induces anoikis via a kinase cascade from ROCK to MKK4/MKK7 to JNK, resulting in the pro-apoptotic triple phosphorylation of Bim (right). Inhibitory integrin antibodies such as Cilengitide or FAK inhibitors such as FAK14 can induce the same anoikis signalling pathway

Anoikis has been mainly studied in an artificial system, where cells are detached by trypsinization and prevented from reattaching to polyHEMA-coated plates^20^. This form of anoikis differs from that induced by GT because trypsin at least partially degrades surface integrins. Therefore, we^30^ and others^58^ could not confirm an earlier study^59^ that JNK was involved in trypsinization-induced anoikis. By contrast, we show here for the first time that not only GT but also FAK and integrin inhibition trigger the same Rho-ROCK-MKK4/MKK7-JNK-mediated signalling pathway indicating that it represents a physiological way to induce anoikis (Fig. 8).

The novel anoikis signalling pathway will increase our understanding of diseases caused by excessive cell detachment or the resistance of detached cells to die. Overexpression of FAK has been shown to promote tumour progression and metastasis^60^, which can be counteracted by high levels of RhoA^61^. Moreover, RhoA downregulation is associated with increased breast cancer cell migration and invasion^62^. Thus, RhoA may act as a tumour suppressor not only by restricting tumour cell motility but also by inducing anoikis. Moreover, FAK is an attractive target for anti-cancer therapy as it has recently been proposed for pancreatic cancer^63^.

On the other hand, activation of FAK or integrins may render lung epithelial cells less sensitive to GT-induced anoikis and hence diminish increased epithelial cell permeability and subsequent lung invasion during *A. fumigatus* infections. An even better strategy is to develop GT inhibitors, which would prevent integrin inactivation and thereby completely block lung epithelial cell anoikis.

## Methods

### Reagents and inhibitors

The ROCK inhibitors Y-27632 (Rho Kinase inhibitor VI) and H-1152 were obtained from EMD Millipore (Billerica, MA, USA); Q-VD-OPh from MP Biomedicals (Eschwege, Germany); and GT from AppliChem (Darmstadt, Germany). The CNFy toxin (150 ng/ml) from *Yersinia pseudotuberculosis* and the C2I/C3 fusion toxin (*Clostridium botulinum* and *Clostridium Limosum*, respectively) combined with the C2II toxin from *Clostridium botulinum* (collectively termed C3 toxin) was produced and purified as described^33,34^. C3 toxin is a binary toxin consisting of C2II (200 ng/ml) and C2I/C3 (100 ng/ml) mixed in cell culture media. The FAK inhibitor 14 (FAK14) was purchased from Tocris (Bristol, UK), and Cilengitide, a cyclic pentapeptide RGD compound (cyclo-[RGDfN(Me)V])^46^, the RGD peptide, DTT and iodocetamide were from Sigma (Taufkirchen, Germany).

### Plasmids

shRNA against *ROCK1* (SHCLNG-NM_005406.1, *shROCK1*: CCGGGCACCAGTTG TACCCGATTTACTCGAGTAAATCGGGTACAACTGGTGCTTTTTG), hu-*CASPASE-8* (SHCLNG-NM_033356.3, *shCASP-8*: CCGGACATGAACCTGCTGGATATTTCTCGAGAAATATCCAGCAGGTTCATGTTTTTTG), hu-*EGFR* (SHCLNV NM_005228.3, *shEGFR*: CCGGGCCTATCAAGTGGATGGCATTCTCGAGAATGCCATCCACTTGAT AGGCTTTTTTG) and a control scrambled shRNA (SHC002 MISSION pLKO.1-puri, *shCTRL*: CCGGCAACAAGATGAAGAGCACCAACTCGAGTTGGTGCTCTTCATCTT GTTGTTTT) were purchased from Sigma. These shRNA were provided in the pLKO.1puro backbone for lentiviral transduction. *Paxillin*-pEGFP was a gift from Rick Horwitz, University of Virginia, USA (Addgene plasmid # 15233). Lentiviral pCDH-EF1a-MCS-T2A-copGFP-empty vector and WT FAK. (exo-FAK) and myristoylated FAK (myrFAK) were kindly provided by Andrew Gilmore, University of Manchester, UK. The endosomal marker mRuby-Endo-14 was a gift from Michael Davidson (Addgene plasmid # 55859).

### Cells and cell treatments

3T9 MEFs either deficient for *Mkk4*, *Mkk7* or both as well as control WT littermates were isolated from respective knockout mice^64,65^. Human bronchial epithelial cells (BEAS-2B) were obtained from American Type Culture Collection (ATCC^®^ CRL-9609^TM^). Cells were cultured in Dulbecco’s modified Eagle’s medium or RPMI supplemented with 10% foetal calf serum (FCS) and 1% penicillin/streptomycin, respectively. BEAS-2B cells were kept subconfluent and only used until passage number 20. Typically, 1 × 10^6^ cells were seeded in 10 cm plates to reach a confluency of approximately 60–70% on the day of the experiment. Murine suspension cells BAF3 (ACC300, DSZM Germany), FL5.12 (Thermo Fisher, Darmstadt, Germany) and human Jurkat T cells (Clone E61, ATCC^®^ TIB152^TM^) were maintained at cell densities lower than 500 000 cells/ml. The former two cell lines were daily supplemented with 1 ng/ml interleukin-3 (Peprotech, Rocky Hill, NJ, USA). Human leukaemia K562 suspension cells (ATCC^®^ CCL243^TM^ were kindly provided by Tilman Brummer (University of Freiburg, Germany). In all, 3 × 10^5^ cells/well were plated in six-well plates for differentiation into megakaryocytes by the addition of 10 ng/ml PMA (Sigma) for 96 h. The medium was replaced daily with fresh medium containing PMA. Anoikis was induced by treating BEAS-2B cells with 1 µM GT for 6 h or 10–50 µM FAK14 for 6–12 h. BEAS-2B cells were also treated with Fc-FasL (20 ng/ml, Adipogen, Epalinges, Switzerland) for up to 4 h to induce extrinsic apoptosis signalling. All cell lines were regularly tested for mycoplasma contamination using a PCR based mycoplasma detection kit (e-MycoTM Mycoplasma PCR Detection Kit, iNtRON Biotechnology Inc., Seongnam, South Korea).

### Attachment of suspension cells

Human fibronectin (Advanced BioMatrix, Carlsbad, CA, USA) was used for the attachment of BAF3 suspension cells. Twelve-well plates were coated with 50 µg/ml fibronectin in phosphate-buffered saline (PBS) overnight. On the next day, 100 000 cells were seeded per well and grown overnight. Non-attached cells were removed by washing with PBS before GT treatment or further analysis.

### Immunoblotting and antibodies

Cell pellets were lysed on ice for 15 min in 50–100 µl whole-cell lysis buffer (20 mM Tris-HCl (pH 7.5), 150 mM NaCl, 5 mM EDTA (pH 8.0), 5 mM Na-pyrophosphate, 1 mM Na_3_VO_4_, 20 mM NaH_2_PO_4_, pH 7.6, 3 mM β-glycerophosphate, 10 mM NaF, 1% phosphatase inhibitor cocktail 1 and 2 (Sigma), 1× protease inhibitor cocktail complete (Roche, Mannheim, Germany) and 20 µM MG-132 (Merck Millipore, Darmstadt, Germany)) containing 1% Triton-X-100. A unit of 40–60 µg of the protein lysate was separated by SDS-polyacrylamide gel electrophoresis (SDS-PAGE) and the proteins were transferred onto nitrocellulose membranes by wet blotting (GE Healthcare Europe, Freiburg, Germany). Membranes were probed for the proteins of interest using the following antibodies: FAK D2R2E (#13009); phospho FAK (pFAK Tyr397) (#8556); paxillin D9G12 (#12065); phospho paxillin (pPaxillin Tyr118) (#2541); MKK4 (#9152); phospho MKK4 (pMKK4 Ser257/Thr261) (#9156); MKK7 (#4172); phospho MKK7 (pMKK7 Ser271/Thr275) (#4171); JNK (#9252); phospho JNK (pJNK Thr183/Tyr185) (#9251); Bim C34C5 (#2933); cleaved caspase-3 Asp175 (#9661); human caspase-8 (D35G2, #4790); PARP (#9542); MYPT1 D6C1 (#8574); phospho MYPT1 (pMYPT1 Thr696) (#5163); integrin β3 (#4702); integrin αV D2N5H (#60896); integrin β1 D6S1W (#34971); P130Cas E1L9H (#13846); and phospho P130Cas (pP130Cas Tyr410) (#4011) (all used at dilutions 1:1000) were bought from Cell Signalling Technology (Danvers, MA, USA). Anti-p190RhoGAP (NB100-88154) and anti-phospho p190RhoGAP (pP190RhoGAP Tyr1105) (NB100-92688) (both 1:1000) were obtained from Novus Biologicals (Littleton, CO, USA); anti-E-cadherin (clone 36/E, 1:2000) from BD Biosciences (Heidelberg, Germany); anti-human EGFR (AF231, 1:2000) from R&D Systems (Wiesbaden, Germany); and mouse monoclonal IgG1 isotype control antibodies (1:1000) from Sigma. Anti-RhoA 26C4 (sc-418, 1:1000) was purchased from Santa Cruz Biotechnology (Dallas, TX, USA). Rat monoclonal anti-tubulin clone YL1/2 (#MCA77G, 1:20000) was obtained from Bio-Rad AbD Serotec (Puchheim, Germany) and mouse monoclonal anti-actin clone C4 (#691000, 1:40000) was purchased from MP Biomedicals SARL (Illkirch-Graffenstaden, France). Polyclonal rabbit antibodies against phospho Bim (pBim) were custom made against a peptide phosphorylated at T112/S114^30^, affinity purified and used at 1:500. Peroxidase-conjugated secondary antibodies against mouse, rabbit or rat IgG (Jackson ImmunoResearch Europe, Suffolk UK) were used for immunodetection (each at 1:2000) using Enhanced Chemiluminescence (Pierce, Rockford, IL, USA) for development. For protein quantitation blots were developed on FusionSL Vilber Lourmat (PeqLab, Erlangen, Germany) and quantified using the FusionCapt Advance Solo 4 (V.16.08) software. Uncropped scans of the western blots are presented in Supplementary Fig. 10.

### Generation of monoclonal anti-GT antibodies

Monoclonal mouse antibodies (mAb) were generated against a fusion protein between GT and the outer surface protein C (OspC) from *Borrelia burgdorferi*. A unit of 100 μg GT was incubated with 3 mg *p*-maleimidophenyl isocyanate (Thermo Fisher) in 500 μl dimethyl sulphoxide for 1 h at room temperature. In parallel 1 mg OspC was allowed to react with 100 μg *N*-succinimidyl *S*-acetylthioacetate (Thermo Fisher) in 500 μl of buffer A (50 mM sodium phosphate, 150 mM NaCl and 1 mM EDTA, pH 7.8). The two solutions were combined, 1.5 ml of buffer A (pH 6.8) was added and the mixture was incubated for another 2 h at room temperature, then buffered to pH 7.3 in PBS. Eight- to 12-week-old Balb/c mice were intraperitoneally immunised three times (every 3–4 weeks) with 20–50 μg GT-OspC antigen emulsified in 200 μl ABM-S complete adjuvans A313 (Linaris, Wertheim-Bettingen, Germany). The fourth immunisation was performed with 50 μg antigen in 200 μl PBS. Three days later mice were sacrificed and the spleen was removed. Spleen cells were isolated and fused with PAI mouse plasma myeloma cells in a ratio 6:1 using polyethylene glycol 1500 (Roche Diagnostics, Risch, Switzerland) before adding 80% RPMI 1640/20% Medium 199, 2 mM l-glutamine and 10% FCS Myoclone plus. The resulting hybridoma cells were distributed in 96 microtiter culture plates and selected on 10^5^/ml thymocyte feeder cells in RPMI/Medium 199 supplemented with hypoxanthine/thymidine (Sigma). The cells were screened for specific IgG production between 2 and 3 weeks post fusion by enzyme-linked immunosorbent assay. Identification of antibody subclasses was performed using a Mouse Monoclonal Antibody Isotyping Kit (Southern Biotech, Birmingham AL, USA). For large-scale mAb production hybridoma cell lines were cultured in a bioreactor (Miniperm, Sarstedt, Nümbrecht, Germany). MAbs were purified by affinity chromatography using protein G Sepharose (GE Healthcare Life Sciences, Freiburg, Germany).

### Anti-GT immunoprecipitations

A volume of 500 µl (1 mg) of a TX-solubilized whole-cell extract from BEAS-2B cells treated with GT for 30 min was pre-cleared with 100 µl of a 50% slurry of Protein G Sepharose^TM^ 4 Fast Flow recombinant protein G beads (GE Healthcare Life Sciences) on a turning wheel at 4 °C for 1 h. After incubating the supernatants with 100 µl of purified mouse mAb anti-GT antibodies (100 µg, clone GT-2.2, IgG1) for 1 h, 50 µl of 50% Protein G Sepharose beads were added and the mixture rotated at 4 °C for 2 h. All beads were centrifuged at 8200 × *g*, 4 °C for 3 min, washed three times with lysis buffer and immune complexes eluted by boiling in Lämmli buffer. The eluted samples were run on non-reducing SDS-PAGE and subjected to anti-integrin αV D2N5H (#60896, 1:1000), anti-integrin β1 D6S1W (#34971, 1:1000), anti-E-cadherin (clone 36/E, 1:2000) or anti-EGFR (AF231, 1:2000) western blot analysis. The following controls were run in parallel: (i) an IP with anti-IgG1 isotype control antibodies instead of anti-GT; (ii) BEAS-2B cells detached in enzyme-free Cell Dissociation Buffer, treated with 150 μM of RGD peptide for 30 min before adding GT, cell lysis and anti-GT IP; and (iii) total extracts from GT-treated cells, incubated 5 mM DTT for 30 min at 37 °C followed by an incubation with 14 mM iodoacetamide at room temperature before subjecting them to anti-GT IP.

### Cell death assays

Cells were treated with 1 µM GT for 6 h, if not stated otherwise. Both detached and attached cells were harvested and used to quantify apoptosis. If pharmacological inhibitors were used, cells were treated for 30 min with the inhibitors prior to GT treatment. For annexin V-fluorescein isothiocyanate (FITC) staining, cells were washed in annexin V binding buffer (10 mM HEPES, 140 mM NaCl and 2.5 mM CaCl_2_), re-suspended in annexin V binding buffer containing annexin V-FITC and incubated for at least 15 min in the dark. The percentage of apoptotic cells was analysed on a Calibur or LSRII equipment (BD Biosciences). Metabolic activity was measured using the Cell Proliferation Kit 2 (XTT, Sigma) according to the manufacturer’s instructions. Briefly, 6 × 10^3^ BEAS-2B cells were plated in 96-well plates, treated on the next day as indicated and incubated with XTT solution for 4 h at 37 °C before analysis in the Tecan infinite M200 microplate reader. For caspase activity assay, 6 µM Ac-DEVD-AMC fluorogenic caspase-3/-7 substrate (Enzo Life Sciences, Lausen, Switzerland) was added to 10 µl of 20–30 µg lysate and 90 µl caspase activity buffer (100 mM HEPES (pH 7.5) and 10 mM DTT) and immediately measured using the Tecan infinite M200 microplate reader (Männedorf, Switzerland). Increasing fluorescence due to substrate cleavage was monitored in intervals of 2 min for 30 min. The slope of the linear regression was used to determine the relative caspase activity.

### Rhotekin pulldown

GST-Rhotekin-RBD beads (Cytoskeleton Inc., Denver, CO, USA) were used to pull down active Rho-GTP from whole-cell lysates^32^. Samples were adjusted to the same cell numbers and lysed in GST-Fish buffer (10% (v/v) glycerol, 50 mM Tris-HCl (pH 7.4), 100 mM NaCl, 1% (v/v) NP-40, 2 mM MgCl_2_ and 1 mM phenylmethylsulfonyl fluoride). Lysates were incubated with Rhotekin beads for 45 min at 4 °C on an overhead shaker. After washing, the samples were boiled in SDS sample buffer and RhoA was detected on western blots.

### Kinase assays

The KinaseSTAR JNK Activity Assay Kit (BioVision, Milpitas, CA, USA) was used to measure JNK activity after GT treatment. The kit was used as stated in the manufacturer’s protocol. In brief, JNK was pulled down from cell lysates and incubated with recombinant c-Jun. Phosphorylation of c-Jun was quantified by western blots. ROCK activity was determinded by quantifying the phosphorylation of its substrate MYPT1 after anti-phospho MYPT1 immunoblotting.

### Site-directed mutagenesis of FAK

To generate superFAK and myr-superFAK, which are activated forms of FAK mutated in their kinase activation loop (K578E/K581E)^39^, we performed site-directed mutagenesis on the lentiviral WT FAK and myrFAK plasmids obtained from Andrew Gilmore^38^ using the QuikChange II XL site-directed mutagenesis kit (Agilent, Santa Clara, USA). The following primers were designed using the Agilent QuikChange Primer Design Tool: FAK K578/581E_fwd: CCATCCATTTAATAGGTAATTTTCCCTC GGAAGCCTCATAGTAAGTACTGTCTTCCATATATCGAGATAA; FAK K578/581E_rev: TTATCTCGATATATGGAAGACAGTACTTACTATGAGGCTTCCGAGGGAAAATTACCTATTAAATGGATGG.

### Lentiviral and retroviral transductions

In all, 2 × 10^6^ HEK 293T cells were transfected with 3 µg of the plasmid of interest, 3 µg envelope vector pMD2G and 3 µg packing vector pSPAX using Attractene Transfection Reagent (Qiagen, Hilden, Germany). After 12 h protein biosynthesis was augmented using 5 mM butyrate (Sigma). Butyrate was removed after 8 h and replaced with 4 ml full medium. Lentiviruses were harvested on the next morning and supplemented with 5 µg/ml polybrene (Sigma). Target cells were spinfected with fresh virus for 10 min at 450 × *g*. Cells were selected with 4 µg/ml puromycin in case of shRNA-mediated knockdown or used without selection for the overexpression of WT (exo-) FAK, myrFAK, superFAK and myr-superFAK.

### Integrin detection by FACS analysis

Cells were harvested in PBS-EDTA by scraping and passed through a 40 µm nylon cell strainer (BD Falcon, Heidelberg, Germany) to yield single cells. After washing in FACS buffer (PBS, 5 mM EDTA and 3% FCS) the cells were stained. For the analysis of integrin expression, cells were incubated for 15 min in 100 µl FACS buffer with the following integrin antibodies (1:100 each): anti CD62-FITC (integrin β3) clone VI-PL2 (#336403); FITC mouse IgG control (#400109) (Bio Legend, Fell, Germany); anti-mouse CD29-FITC (integrin β1, # MCA2298F); anti-active integrin β1 12G10 (Alexa Fluor^®^ 488, ab202641); or hamster IgG negative control-FITC (# MCA2356F) (Bio-Rad AbD Serotec).

Cyclo-RGD-5-FAM (#65160) (AnaSpec, MoBiTech, Göttingen, Germany) was used to assess integrin ligand-binding activity. The RGD peptide represents the integrin-binding motif in fibronectin and vitronectin. Active integrins can therefore bind to the fluorescently labelled peptide and be quantified. Collected cells were washed in FACS buffer and subsequently stained with 2.5 µM Cyclo-RGD-5-FAM in 200 µl FACS buffer for 15 min before analysis.

### Integrin inhibition

BEAS-2B cells were detached in Cell Dissociation Buffer, enzyme-free (Thermo Fisher Scientific) to prevent degradation of surface molecules. Cells were either suspended in medium alone, medium containing 25 µg/ml Cilengitide (Sigma) or medium containing 100 µg/ml of the inhibitory anti-integrin β1 (clone P4C10, MAB1987Z, Merck Millipore) or 25 µg/ml of the inhibitory anti-integrin α5β1 (clone JBS5, MAB1969, Merck Millipore) antibodies or both and incubated for 0–24 h. At different time points cells or cellular extracts were subjected to survival (metabolic) and caspase-3 activity assay, western blot analysis or phase contrast microscopy analysis.

### Microscopy and immunofluorescence analysis

Brightfield micrographs were taken with the Nikon eclipse TS100 inverted microscope (Düsseldorf, Germany), equipped with DS-L3 controller for image acquisition. For live cell imaging, 6 × 10^4^ BEAS-2B cells were seeded in glass-bottom dishes (Greiner Bio One, Frickenhausen, Germany) and transfected with 5 µg GFP-paxillin and mRuby-Endo-14 using Lipofectamine 2000 reagent (Thermo Fisher Scientific). Cells were analysed 12 h post transfection.

For immunfluorescence analysis, 5 × 10^3^ BEAS-2B cells per well were seeded on IBIDIµ eight-well glass-bottom slides and treated with 1 µM GT on the following day for the indicated time points. The cells were immediately fixed in the well by adding 4% paraformaldehyde (final concentration) and stained as described in Alonko et al.^66^ using anti-pFAK-Y397 (clone 44–624 G, Life Technologies, used 1:150) and anti-EEA1 (clone 14/EEA1, BD Biosciences, #610457, used 1:100) antibodies. The secondary antibodies were goat anti-rabbit Alexa 568-conjugated for pFAK and goat anti-mouse Alexa 488-conjugated for EEA1 (both 1:400 of a 2 mg/ml stock from Invitrogen, Darmstadt, Germany). Confocal fluorescence microscopy was performed with an inverted microscope (Axiovert 200M; Carl Zeiss, Jena, Germany) equipped with a spinning-disk head (Yokogawa, Tokio, Japan) with emission filters, and solid-state laser lines (405, 488 and 561 nm). Fluorescence images were collected with a CoolSNAP-HQ2 digital camera (Roper Scientific, Munich, Germany) driven by VisiView imaging software (Visitron Systems, Puchheim, Germany). Glass-bottom dishes were incubated in a humidified atmosphere (6.5% CO_2_ and 9% O_2_) at 37 °C. Images were processed with Metamorph software (Universal Imaging, New York, USA).

### Mass spectrometry

MS was performed to analyse the covalent modification of integrins by GT. Human recombinant αVβ3 integrin (5 µg) was mixed with 5 µg bovine serum albumin as an internal control in 50 mM HEPES, pH 7.5, incubated with 100 μM to 1 mM GT at room temperature for up to 6 h and then denatured in 0.1% RapiGest (Waters, Eschborn, Germany) for 45 min at 70 °C. Peptides were generated by digestion with 0.2 µg trypsin overnight at 37 °C and then purified using C18 STAGE solid phase extraction columns (Varian, Palo Alto, CA, USA). A Q-Exactive Plus Hybrid Quadrupole-Orbitrap system (Thermo Scientific, Darmstadt, Germany) was used for mass spectrometry and operated in the data-dependent mode^67^. Peptide sequences were identified by X! TANDEM (Version 2013.09.01) in conjunction with PeptideProphet using the reviewed canonical human (20272 protein sequences) and bovine (5994 proteins) combined sequence database, downloaded from Uniprot on 26.11.2013 and 15.07.2015 respectively, together with an equal number of randomised decoy sequences, generated by DBtoolkit. The PeptideProphet minimum probability threshold was set to 0.05. The GT modification of cysteines was calculated as an adduct with a variable modification with a mass difference of 326.0395 Da. Only peptides with a probability score > 0.99 were taken into consideration. Identified peptides were mapped on the protein sequence using the software Proteator.

### Integrin Affymetrix microarray and RNAseq analyses

To quantify the expression of integrin genes, raw data of the transcriptome of untreated Beas-2B cells from three high-throughput experiments, one microarray and two RNAseq assays data were downloaded from Gene Expression Omnibus and Array Express with respective series/sample IDs GSE24025/GSM591439 as well as E-MTAB-4729/ERR1406031 and ERR1406032.

Affymetrix Human Gene 1.0 ST microarray was normalised using Single-Channel Array^68^ and exon expression was summarised to the gene level using the R/Bioconductor package pd.hugene.1.0.st.v1 (version 3.14.1). Transcripts were considered to be present in the cell if they have an absolute expression value > 0. RNAseq data were pseudoaligned to the cDNA transcript sequences Ensembl version 87 and quantified using kallisto (Version 0.43.1)^69^. Transcripts abundance was summarised to the gene level via the R library tximport^70^. Genes were considered to be expressed, if they have a minimal transcript per million value > 1.

## Electronic supplementary material

Supplementary Information

Description of Additional Supplementary Files

Supplementary Movie 1

## Data Availability

All data supporting the findings of this study are available within the article and its supplementary information files or from the corresponding author upon reasonable request
