# Supplementary material for: Identification of a novel anoikis signalling pathway using the fungal virulence factor gliotoxin
Source: Nat Commun. 2018 Aug 30;9:3524. doi: 10.1038/s41467-018-05850-w (PMC6117259; doi:10.1038/s41467-018-05850-w)
Supplement: Supplementary file 1 — Supplementary Information [file 41467_2018_5850_MOESM1_ESM.pdf]

Supplementary Information

Identification of a novel anoikis signalling pathway using the fungal virulence factor gliotoxin

by Haun, F. et al.

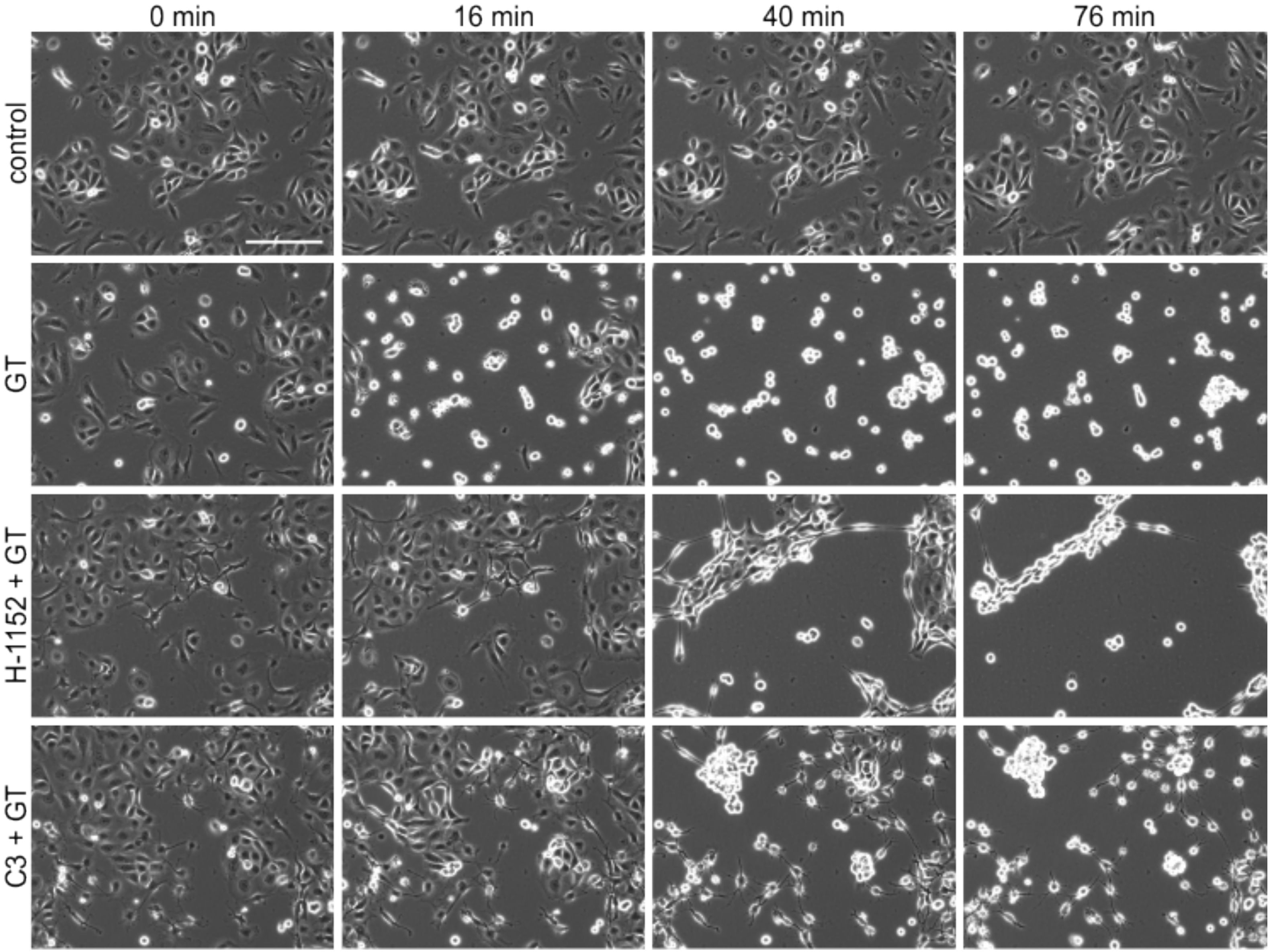

Supplementary Figure 1: Lung epithelial cells rapidly detach in response to GT. Phase contrast microscopy showing the rapid GT-induced detachment of human bronchial epithelial cells (BEAS-2B) within 75 min. In the presence of the 1 μM of the ROCK inhibitor H-1152 or 100 ng/ml of the RhoA inhibitor C3 toxin cell detachment is slightly delayed. Scale bar = 200 μm. Images shown are representatives of three independent experiments.

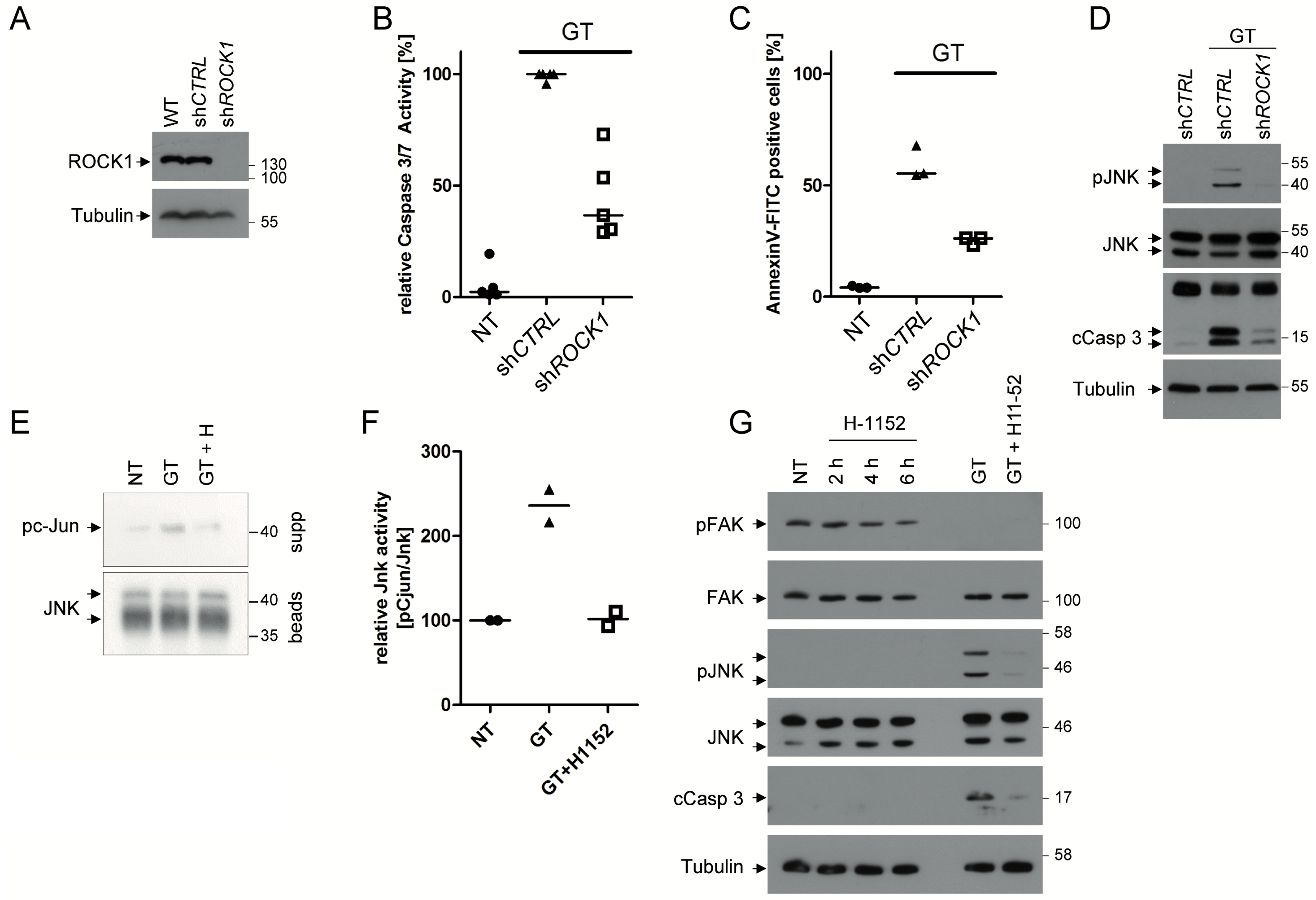

Supplementary Figure 2: *ROCK1* knock-down inhibits that GT-induced anoikis (A) Western blots of total extracts of BEAS-2B cells infected with control shRNA (sh*CTRL*) or shRNA against *ROCK1* (sh*ROCK1*). An uninfected WT is also shown. **(B-D)** sh*ROCK1* BEAS-2B cells showed reduced caspase-3/7 activation (B), degree of apoptosis (C), JNK phosphorylation and caspase-3 processing (D) after 1 µM GT treatment for 6 h as compared to sh*CTRL* cells. **(E)** JNK activity assay. Anti-phospho-c-Jun (pc-Jun) and anti-JNK western blots showing that rec. c-Jun can be *in vitro* phosphorylated in an anti-JNK IP from extracts of GT-treated BEAS-2B cells, but not if the cells were pre-treated with 1 µM of the ROCK inhibitor H-1152. **(F)** Lumi Imager quantification of the chemiluminescent immunoblot bands shown in (E). **(G)** Western blots of total extracts of BEAS-2B cells either untreated (NT) or treated with 1 µM of H-1152 for 2-6 h or treated with 1 µM GT ± H-1152 for 2 h showing that inhibition of ROCK by H-1152 does not prevent GT-induced dephosphorylation of FAK (pFAK) but blocks JNK phosphorylation and caspase-3 processing (cCasp-3). Tubulin as loading controls. Immunoblots in (A), (D), (G) are representatives of three, the blots shown in (E) of two independent experiments. Graphs in (B), (C) show the means and individual data points of five and three independent experiments, respectively. Graph in (F) shows the mean and individual data points of two independent experiments, normalized to untreated (nt) samples.

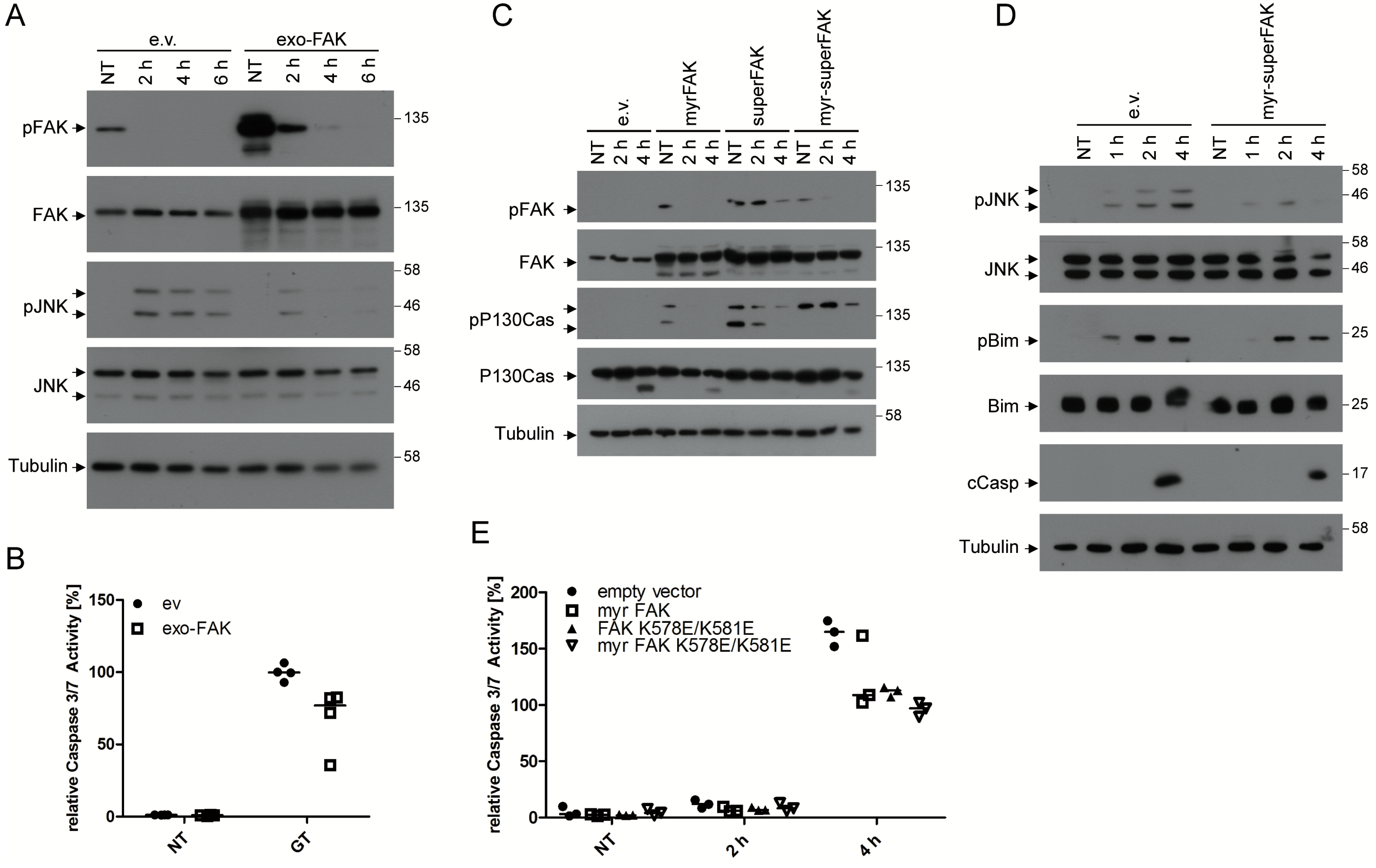

Supplementary Figure 3: Active FAK partially protects from GT-induced anoikis. (A) Western blot analysis of total extracts of BEAS-2B cells showing active phosphorylated (pFAK) exogenous wild-type FAK (exo-FAK) in non-treated (NT) cells. GT induced dephosphorylation of endogenous and overexpressed FAK. However in exo-FAK cells, more phosphorylated FAK remained after 2 h of GT treatment causing a lower JNK phosphorylation than in empty vector (e.v.) cells. **(B)** Higher levels of active exo-FAK reduced caspase-3/-7 activity in BEAS-2B treated for 6 h with 1 µM GT. **(C/D)** Same analysis as in (A) but with BEAS-2B cells infected with either e.v. or active forms of FAK (myrFAK, superFAK (FAK K578E/K581E), myr-superFAK), treated with GT for 1-4 h. FAK mutants were overexpressed at similar levels, phosphorylated at Y397 and able to phosphorylate the p130Cas substrate. GT treatment caused FAK and p130Cas dephosphorylation, JNK phosphorylation (pJNK), Bim phosphorylation (pBim) and caspase-3 processing (cCasp). All events were delayed in active FAK mutants as compared to e.v. controls, esp. in myr-superFAK. **(E)** Overexpression of activated FAK mutants reduced caspase-3/-7 activity (DEVDase) in BEAS-2B treated with 1 µM GT for 2-4 h. Tubulin as loading control in (A), (C), (D). Immunoblots in (A) are representatives of four experiments, blots in (C), (D) are representatives of three independent experiments. Graphs in (B), (E) show the means and individual data points of four and three independent experiments, respectively.

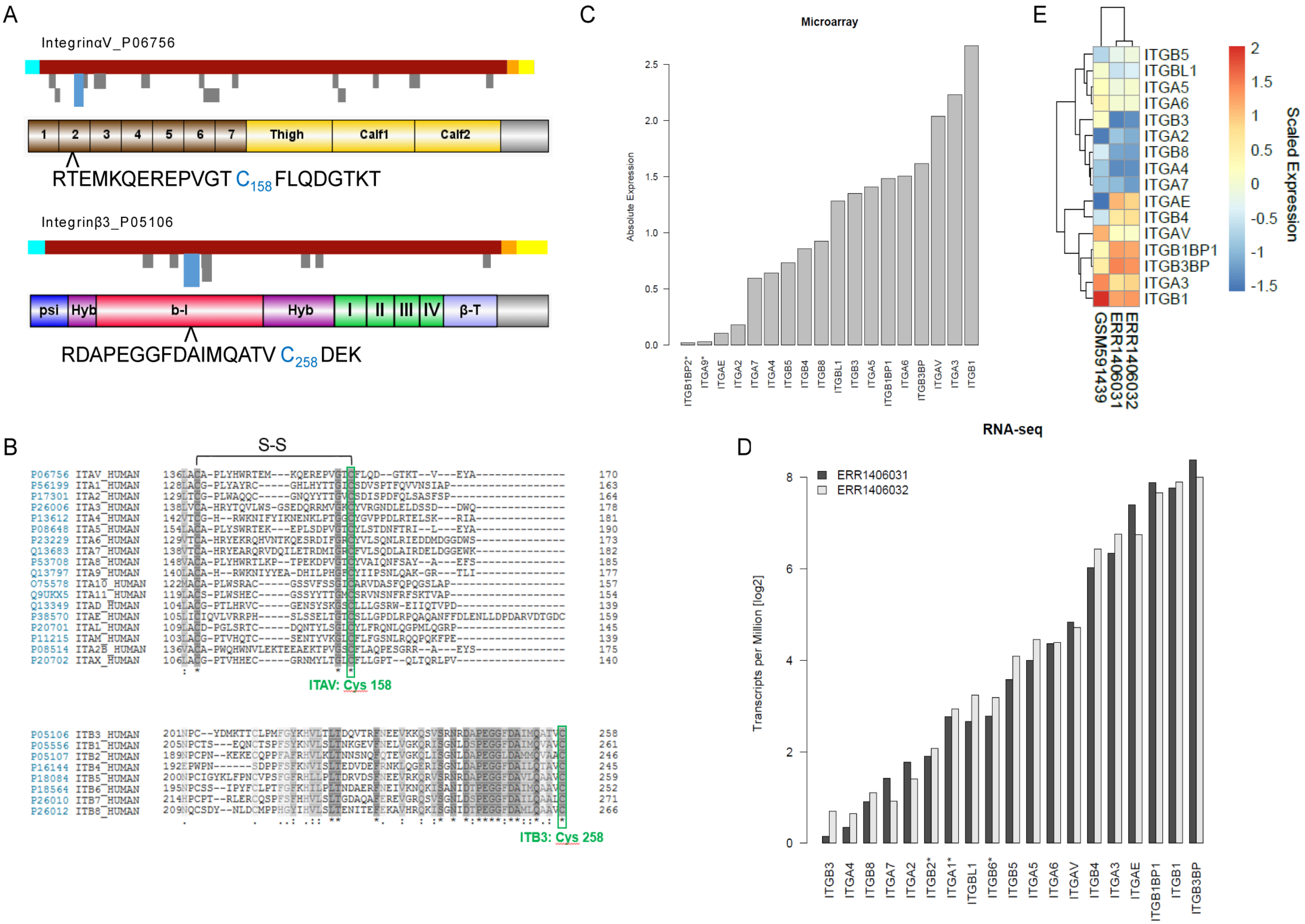
A
IntegrinαV_P06756
1 2 3 4 5 6 7 Thigh Calf1 Calf2
RTEMKQEREPVGT C158 FLQDGTKT
Integrinβ3_P05106
psi Hyb b-I Hyb I II III IV β-T
RDAPEGGFDAIMQATV C258 DEK
B
S-S
P06756 ITAV_HUMAN
P56199 ITA1_HUMAN
P17301 ITA2_HUMAN
P26006 ITA3_HUMAN
P13612 ITA4_HUMAN
P08648 ITA5_HUMAN
P23229 ITA6_HUMAN
Q13683 ITA7_HUMAN
P53708 ITA8_HUMAN
Q13797 ITA9_HUMAN
O75578 ITA10_HUMAN
Q9UKX5 ITA11_HUMAN
Q13349 ITAD_HUMAN
P38570 ITAE_HUMAN
P20701 ITAL_HUMAN
P11215 ITAM_HUMAN
P08514 ITA2B_HUMAN
P20702 ITAX_HUMAN
ITAV: Cys 158
P05106 ITB3_HUMAN
P05556 ITB1_HUMAN
P05107 ITB2_HUMAN
P16144 ITB4_HUMAN
P18084 ITB5_HUMAN
P18564 ITB6_HUMAN
P26010 ITB7_HUMAN
P26012 ITB8_HUMAN
ITB3: Cys 258
C
Microarray
Absolute Expression
ITGB1BP2* ITGA9* ITGAE ITGA2 ITGA7 ITGA4 ITGB5 ITGB4 ITGB8 ITGBL1 ITGB3 ITGA5 ITGB1BP1 ITGA6 ITGB3BP ITGAV ITGA3 ITGB1
D
RNA-seq
ERR1406031
ERR1406032
Transcripts per Million [log2]
ITGB3 ITGA4 ITGB8 ITGA7 ITGA2 ITGB2* ITGA1* ITGBL1 ITGB6* ITGB5 ITGA5 ITGA6 ITGAV ITGB4 ITGA3 ITGAE ITGB1BP1 ITGB1 ITGB3BP
E
ITGB5 ITGBL1 ITGA5 ITGA6 ITGB3 ITGA2 ITGB8 ITGA4 ITGA7 ITGAE ITGB4 ITGAV ITGB1BP1 ITGB3BP ITGA3 ITGB1
GSM591439 ERR1406031 ERR1406032
Scaled Expression

Supplementary Figure 4: GT modifies cysteines in the RGD binding region of integrins (A) Recombinant human integrin αVβ3 (5 µg) was incubated with 1 mM GT for 6 h. After trypsin digestion the resulting peptides were analyzed by Q-Exactive Plus mass spectrometry, identified by the Trans-Proteomic Pipeline (TPP), retrieved from Uniprot and mapped to the respective integrin protein sequences using the software Proteator. Identified peptides are marked in grey, those with an additional mass corresponding to GT in blue. The αV chain bound GT at Cys158 in the seven blade β-propeller domain (brown). The β3 chain was modified by GT at Cys258 in the ligand binding domain (β-I, red). Domain structures were drawn using the Illustrator for Biological Sequences (IBS V1.0, not in scale). PSI: plexin-semaphorin-integrin domain, Hyb: Hybrid domain, I-IV: I-EGF domains 1 to 4, β-T: beta tail. **(B)** Protein sequences from different integrin α (top) and β chains (bottom), retrieved from Uniprot and aligned using Clustal Omega (V1.2.1). Cys158 (relative to human αV, green box) was conserved in all alpha subunits. This cysteine forms a disulphide bridge with Cys138. The Cys258 of the β3 subunit (green box) is also conserved and bonds with Cys299 (not shown). Disulphide linkages were first described elsewhere[43,44]. **(C-E)** BEAS-2B cells express 14 integrin genes. (C) Barplots depicting the absolute expression of integrin genes in BEAS-2B based on an Affymetrix Human Gene 1.0 ST Array obtained from Gene Expression Omnibus (sample ID GSM591439). (D) Barplots showing the transcript per million values from two RNAseq samples from ArrayExpress (IDs E-MTAB-4729/ERR1406031 and ERR1406032). Genes that are found exclusively in either data are marked with an asterisk. (E) Heatmap depicting the scaled expression of all commonly expressed integrin genes in the two RNAseq and the one microarray datasets. Expression values were scaled sample-wise to have zero mean and a standard deviation. Rows and columns are hierarchically clustered according to their Euclidean distances using complete linkage. All samples show good agreement in expression with the strongest expression for ITGB1 (Integrin β1), ITGA3 (Integrin α3) and ITGAV (Integrin αV).

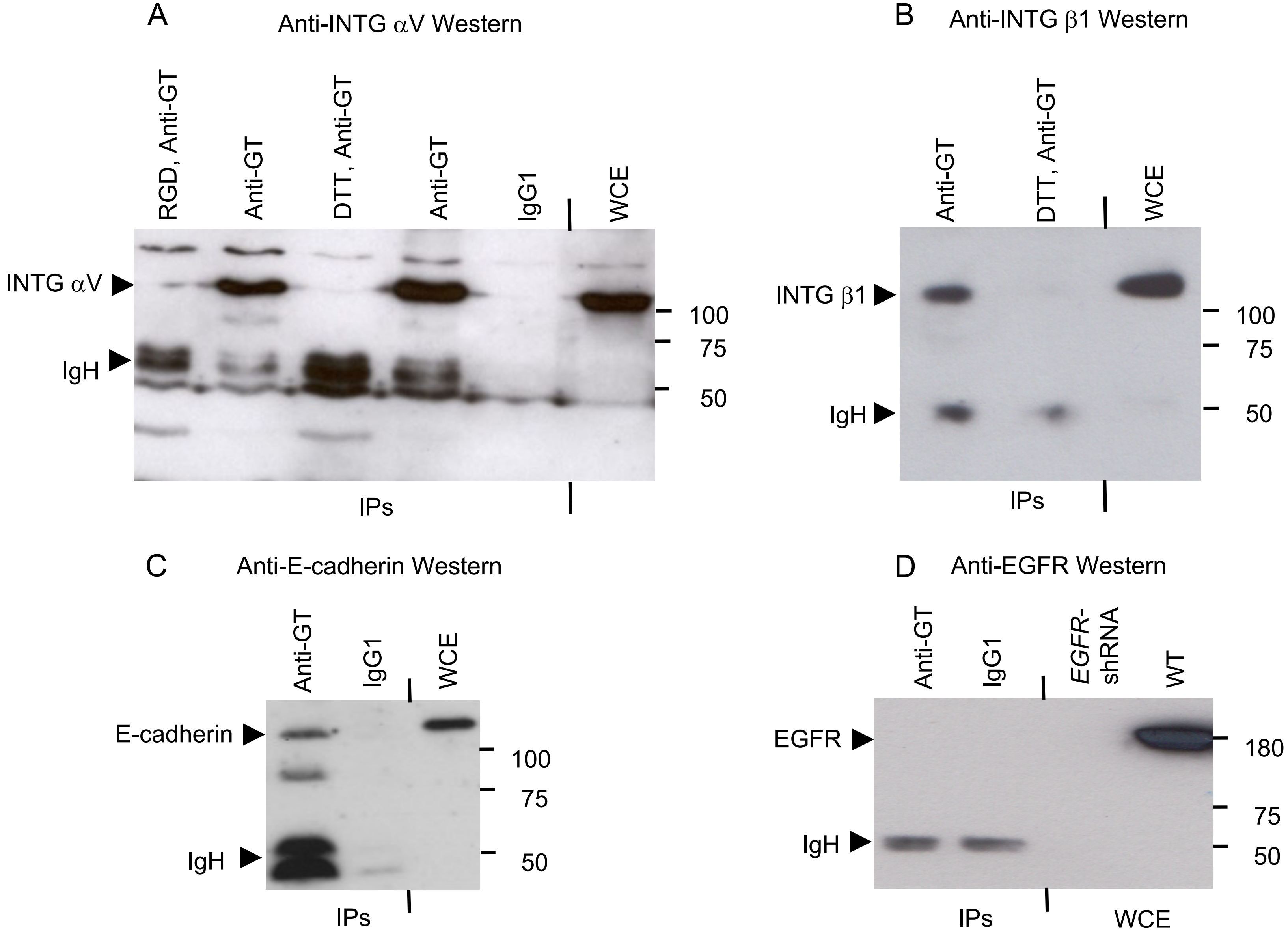

Supplementary Figure 5: Integrins and E-cadherin are found in anti-GT co-IPs. (A) Anti-integrin (INTG) αV, **(B)** anti-integrin β1, **(C)** anti-E-cadherin or **(D)** anti-EGFR western blots of anti-GT or anti-IgG1 control IPs of whole cell extracts (WCE) of BEAS-2B cells treated with 1 μM GT for 30 min showing a specific immunoprecipitation of integrin chains and E-cadherin dependent on disulphide bridge formation of GT at the RGD binding site of integrins. As additional controls in (A) and (B) the cells were pretreated with RGD peptide before adding GT (RGD, Anti-GT), or total extracts from GT-treated cells were incubated with DTT followed by iodoacetamide before anti-GT IP (DTT, Anti-GT). In (D) a WCE from cells infected with *EGFR* shRNA was compared to that of wildtype (WT) cells to identify the EGFR protein band. IgH: immunoglobulin heavy chains from IPs detected by the secondary antibodies on western blots. Western blots are representative of three independent experiments.

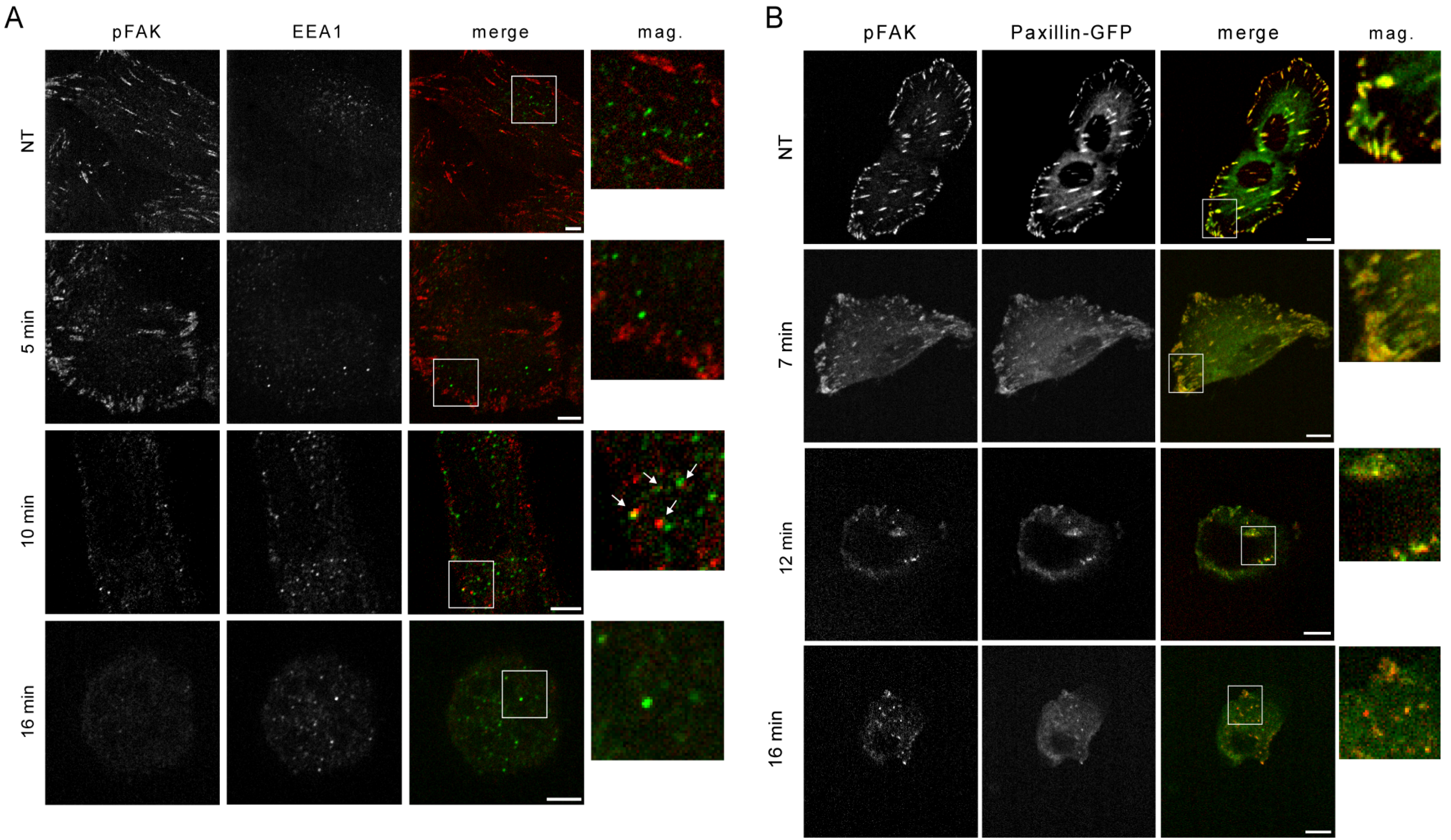

Supplementary Figure 6: pFAK is rapidly dephosphorylated by GT before endocytosed. (A) Phosphorylation and plasma membrane or endosome localization of FAK were studied by confocal video time lapse microscopy in BEAS-2B cells treated with 1 µM GT for 0 (NT) to 16 min. pFAK was visualized with goat anti-rabbit Alexa568, the endosomal marker EEA1 with goat anti-mouse Alexa488 secondary antibodies. In the absence of GT, pFAK was present in focal adhesions (red) and not in endosomes (EEA1, green). After GT treatment, the red fluorescence signal of FAK gradually disappeared and pFAK did not co-localize with EEA1 (green) (arrows) except in rare cases (10 min, yellow staining). **(B)** The same analysis as in (A) but in cells overexpressing paxillin-GFP. In untreated cells pFAK predominantly co-localized with paxillin-GFP in focal adhesions (yellow staining). After GT, paxillin-GFP migrates into endosomes (also see Fig. 4, Supplementary Movie 1). By contrast pFAK was rapidly lost (dephosphorylation) before it co-localized with paxillin-GFP in endosomes. Scale bar = 5 µm, mag. = magnification of the boxed areas in “merge”.

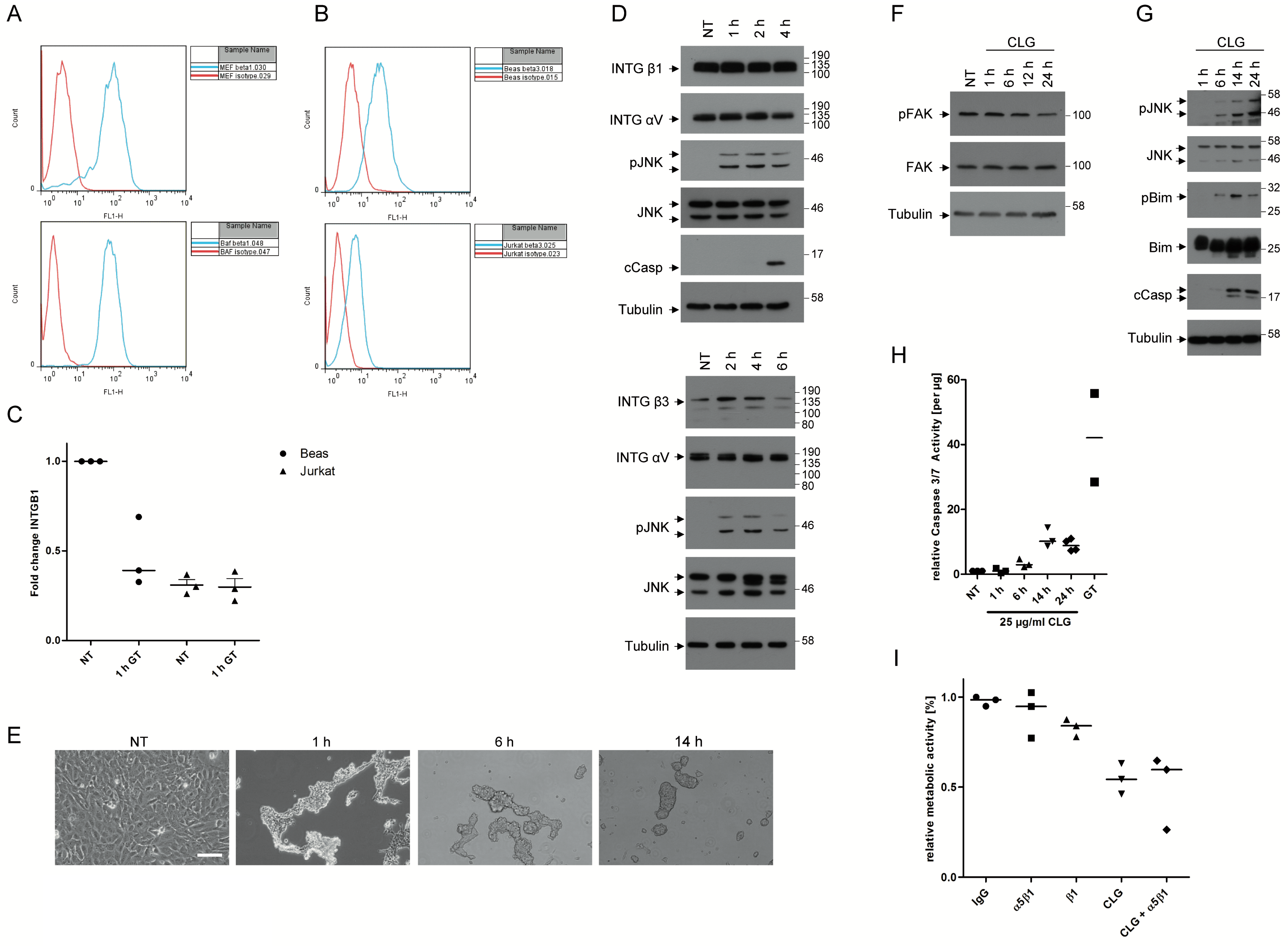
A
Sample Name
MEF beta1.030
MEF isotype.029
Baf beta1.048
BAF isotype.047
Count
FL1-H
B
Beas beta3.018
Beas isotype.015
Jurkat beta3.025
Jurkat isotype.023
C
Beas
Jurkat
Fold change INTGB1
NT
1 h GT
D
NT 1 h 2 h 4 h
INTG β1
INTG αV
pJNK
JNK
cCasp
Tubulin
NT 2 h 4 h 6 h
INTG β3
E
NT
1 h
6 h
14 h
F
CLG
NT 1 h 6 h 12 h 24 h
pFAK
FAK
G
1 h 6 h 14 h 24 h
pBim
Bim
H
relative Caspase 3/7 Activity [per µg]
NT 1 h 6 h 14 h 24 h GT
25 µg/ml CLG
I
relative metabolic activity [%]
IgG
α5β1
β1
CLG
CLG + α5β1

Supplementary Figure 7: Integrin inhibitor Cilengitide activates the same anoikis pathway as GT. Representative histograms of three independent FACS analysis of integrin β1 (MEF, BAF3) **(A)** or integrin β3 (BEAS-2B, Jurkat) **(B)** surface expression using β chain antibodies (blue lines). Respective isotype antibodies as controls (red lines). **(C)** FACS analysis of active integrin β1 surface expression on BEAS-2B versus Jurkat cells using the anti-active integrin β1 antibody 12G10. While BEAS-2B express active integrin β1 on their surface (gray bars) which diminishes after GT treatment, Jurkats show very little integrin expression (black bars). **(D)** Western blot analysis of extracts of BEAS-2B cells showing the expression of integrins αV, β1 and β3. Treatment with 1 μM GT for 1-4 h induces anoikis via JNK phosphorylation (pJNK) and caspase-3 processing (cCasp) but the expression levels of all three integrins remain stable. **(E)** Phase contrast microscopy of BEAS-2B cells treated with 25 μg/ml Cilengitide (CLG) for 0 (NT) to 14 h. Cells rapidly detach but in sheets rather than single cells (compare to GT in Supplementary Fig. 1) (Scale bar = 100 μm). **(F/G)** Western blots of extracts of BEAS-2B cells treated with 25 μg/ml CLG for 1-24 h showing that FAK gets partially dephosphorylated (F) concomitant with JNK phosphorylation (pJNK), Bim phosphorylation (pBim) and caspase-3 processing (cCasp) (G). **(H)** Caspase-3/7 activity assay of extracts of BEAS-2B cells treated with 25 μg/ml CLG for up to 24 h showing a slight increase in caspase-3 activity after 14 h. **(I)** Survival of BEAS-2B cells (measured as relative metabolic activity using the XTT assay) treated with CLG, inhibitory anti-integrin β1 or α5β1 antibodies or an IgG control antibody alone or in combination for 24 h. While CLG can induce anoikis, the inhibitory integrin antibodies were unable to do so. This indicates that CLG triggers the same anoikis pathway as GT but less efficiently. Tubulin as loading control in (D), (F), (G). Western blots shown in (D), (F), (G) as well as images shown in (E) are representatives of three independent experiments. Graphs in (C), (H), (I) show the means and individual data points of three independent experiments. Data in (I) are normalized to the IgG control sample.

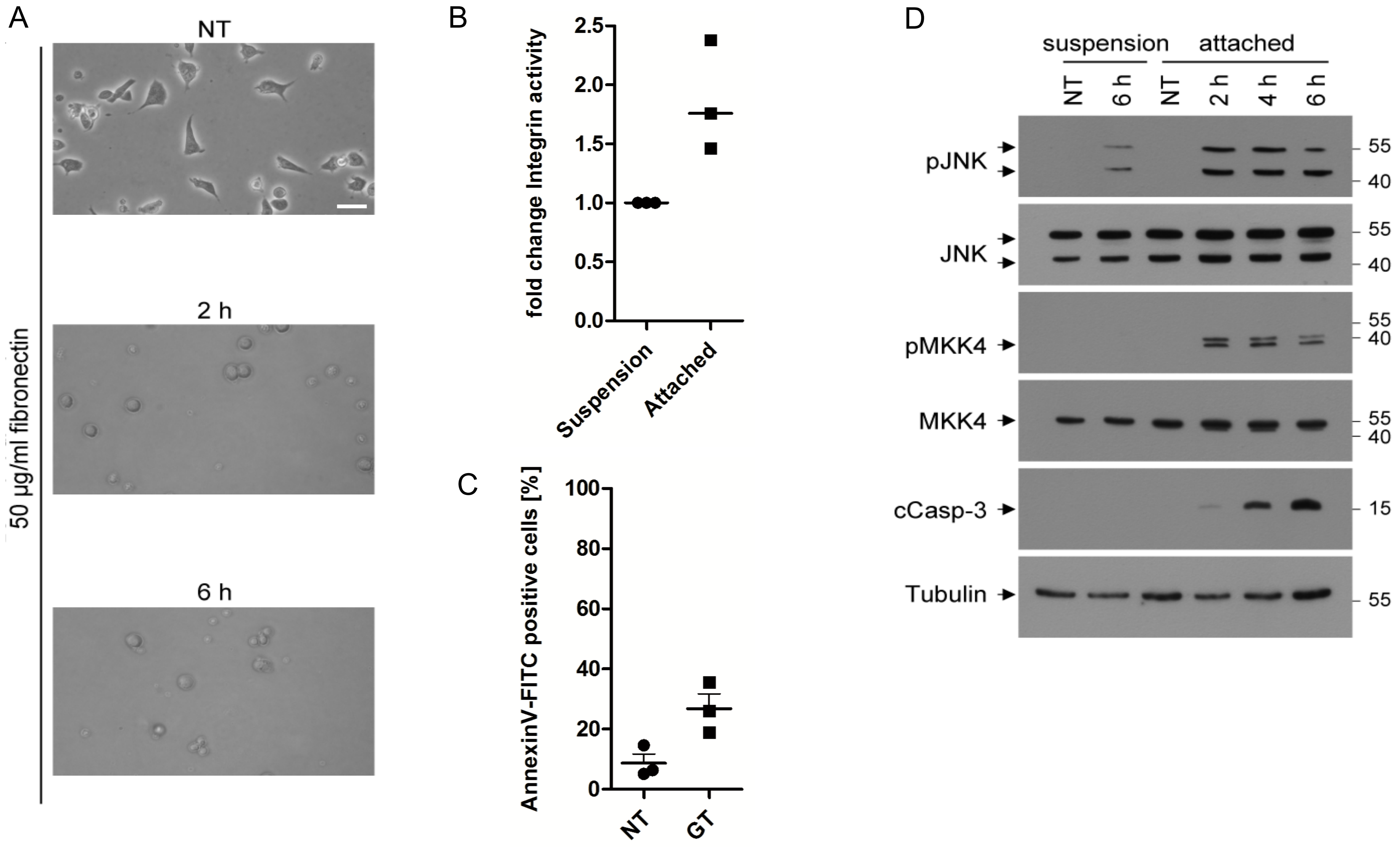

Supplementary Figure 8: Attachment of suspension cells sensitizes to GT-induced anoikis (A) BAF3 suspension cells were grown overnight on fibronectin coated plates (50 µg/ml). Attached BAF3 were challenged with 1 µM GT for indicated time points. Cells detached in response to GT and acquired apoptotic morphology. Scale bar = 100 µm. **(B/C)** Attachment of suspension cells activated integrins, assessed by RGD-FAM FACS staining (B) and sensitized them for GT-induced apoptosis as measured by annexin V-FITC FACS staining (C). **(D)** Western blots of total extracts of suspension or fibronectin-attached BAF3 cells either untreated (NT) or treated with 1 µM GT for up to 6 h showing that while suspension cells do not show MKK4-JNK-mediated caspase-3 activation, attached cells exhibit MKK4 and JNK phosphorylation and caspase-3 processing in response to GT. Tubulin as loading control. Graphs in (B), (C) show the means and individual data points of three independent experiments, data in (B) are normalized to the suspension cell sample. Images in (A) and western blots in (D) are representatives of three independent experiments.

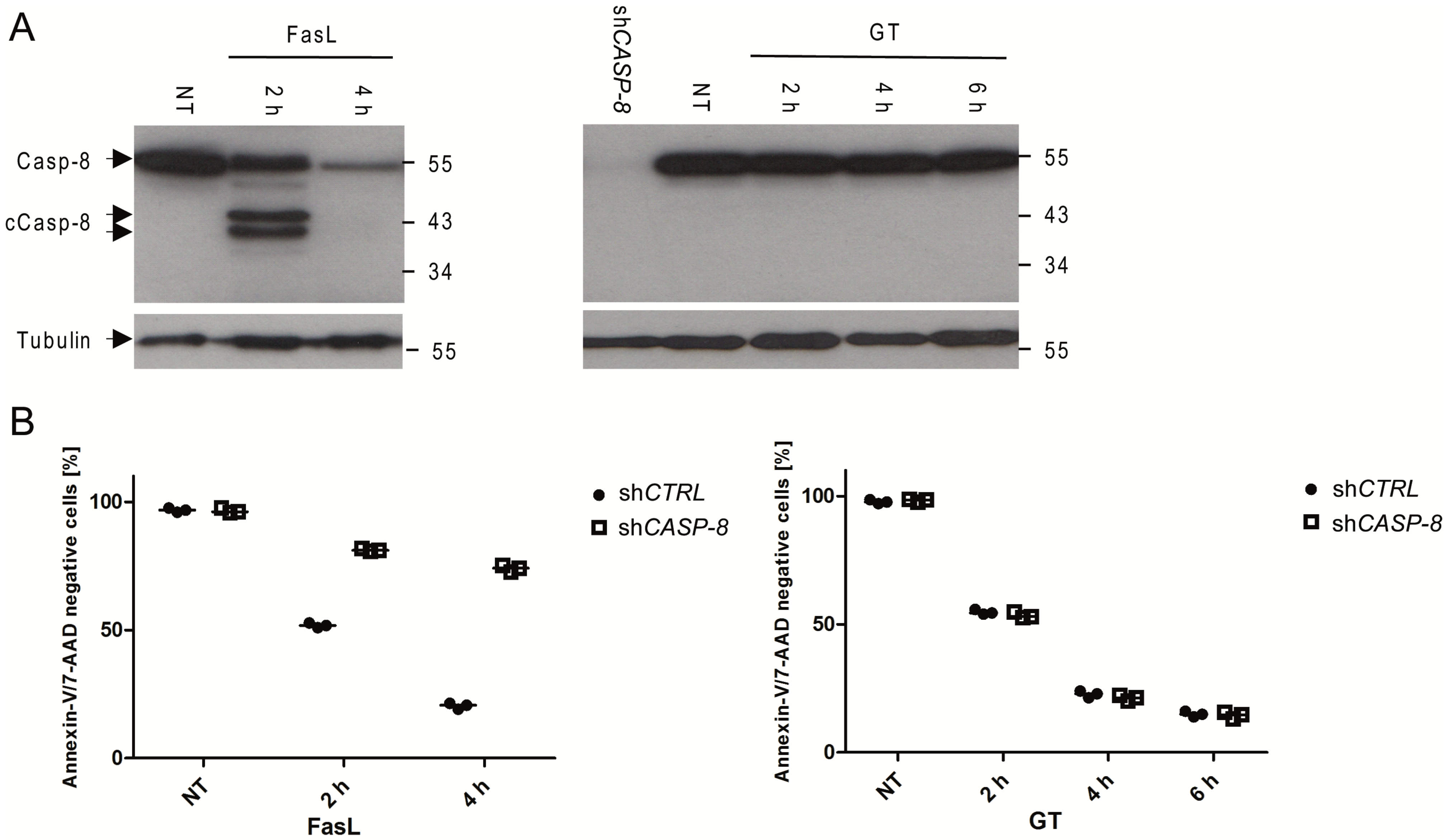

Supplementary Figure 9: Caspase-8 is not required for GT-induced apoptosis. (A) Anti-caspase-8 western blots of total extracts of BEAS-2B cells either untreated (NT), treated with 20 ng/ml recombinant Fc-FasL for 2 and 4 h or treated with 1 µM GT for 2, 4 and 6 h. 51 kD pro-caspase-8 (Casp-8) is processed to 43/44 kD active caspase-8 (cCasp-8) in response to FasL but not GT. Tubulin as loading control. **(B)** BEAS-2B cells deficient for caspase-8 expression by lentiviral transduction of *CASPASE*-8 shRNA (sh*CASP-8*) are more resistant to FasL-induced apoptosis (as determined by annexin-V/PI FACS analysis) but die similarly in response to GT when compared to cells expressing a control shRNA (sh*CTRL*). Western blots shown in (A) are representatives of three independent experiments. Graphs in (B) show the means and individual data points of three independent experiments.

Figure 1 A

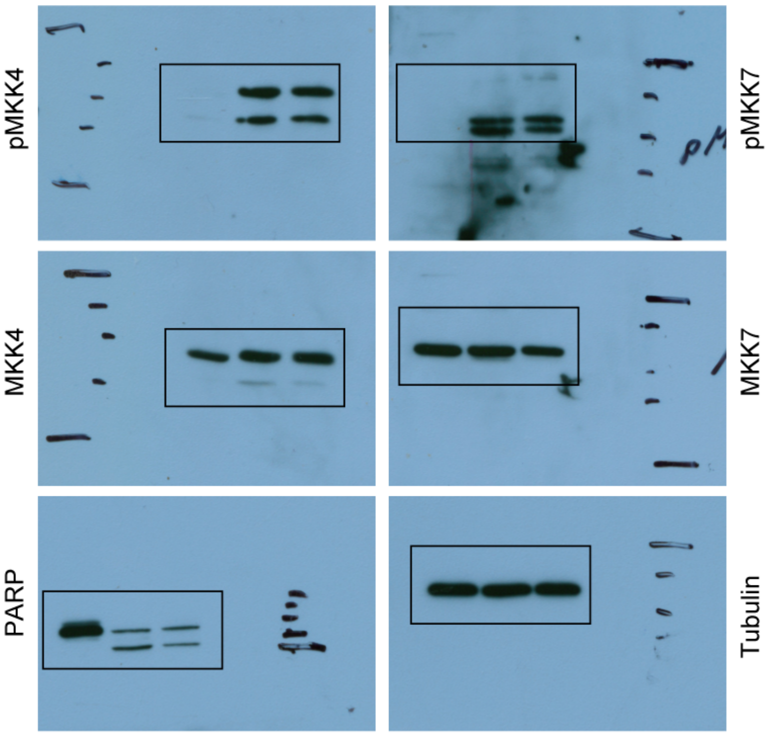

Figure 1 B

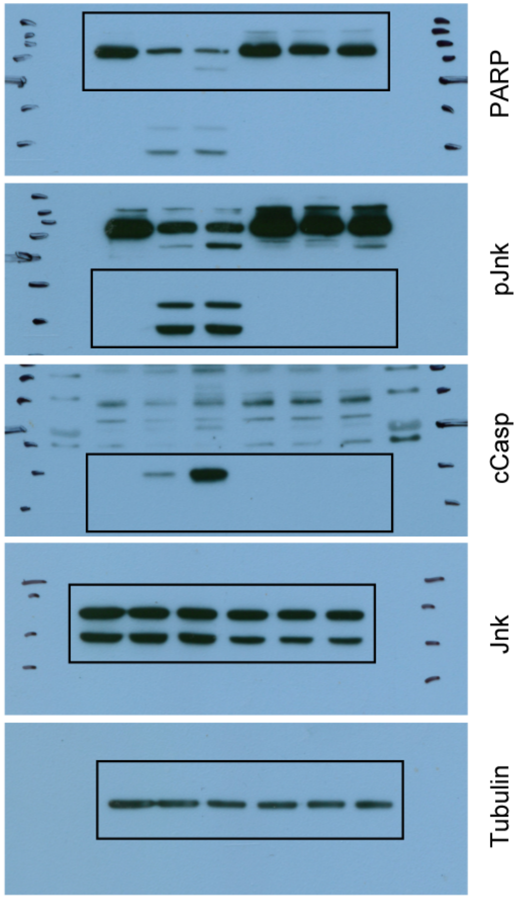

Figure 1 B, lower part

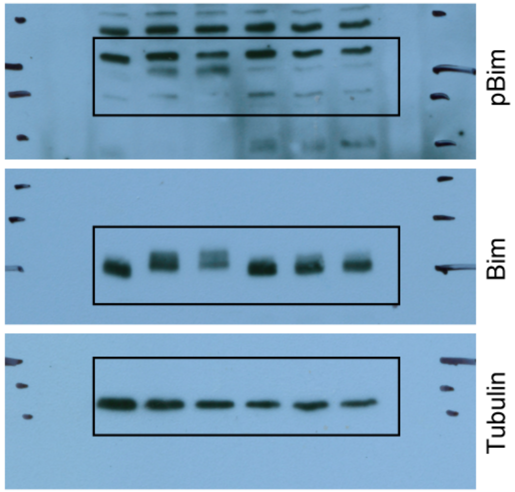

Figure 2 A

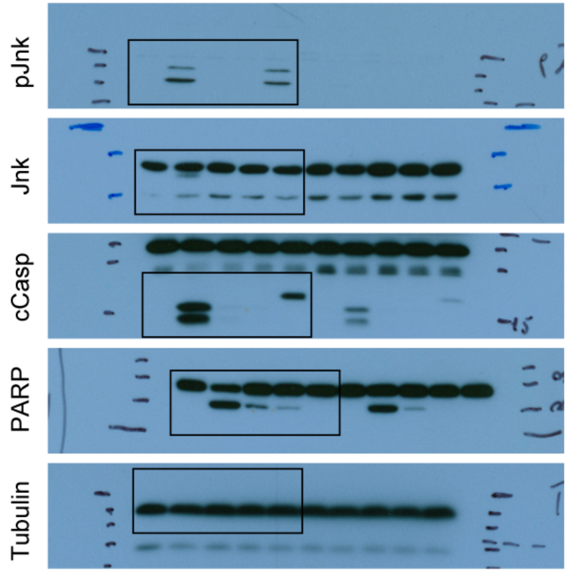

Figure 2 B

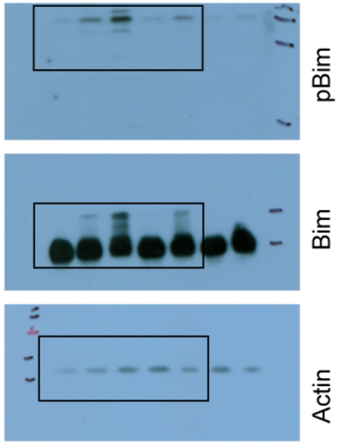

Figure 2 C

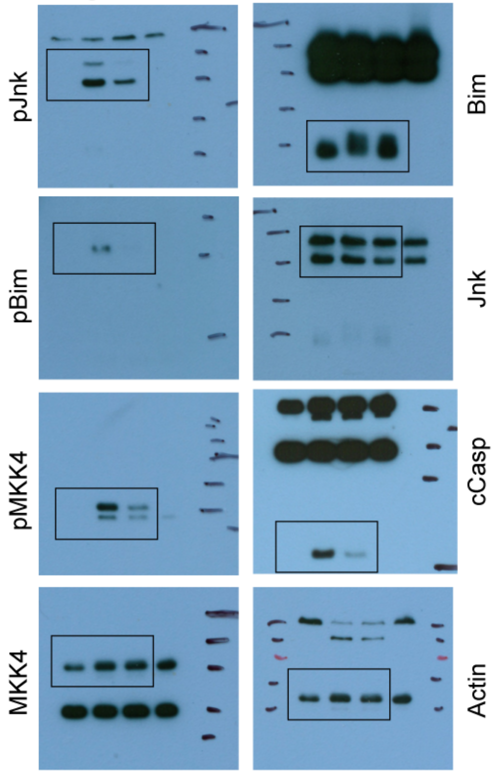

Supp2 E

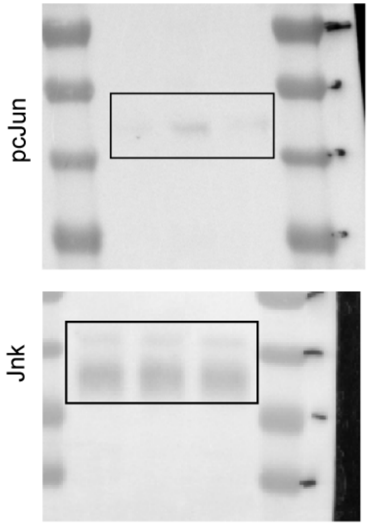

Supp2 A

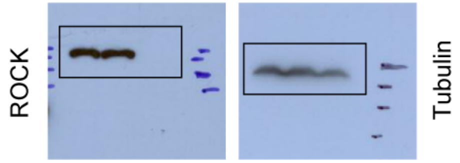

Supp2 D

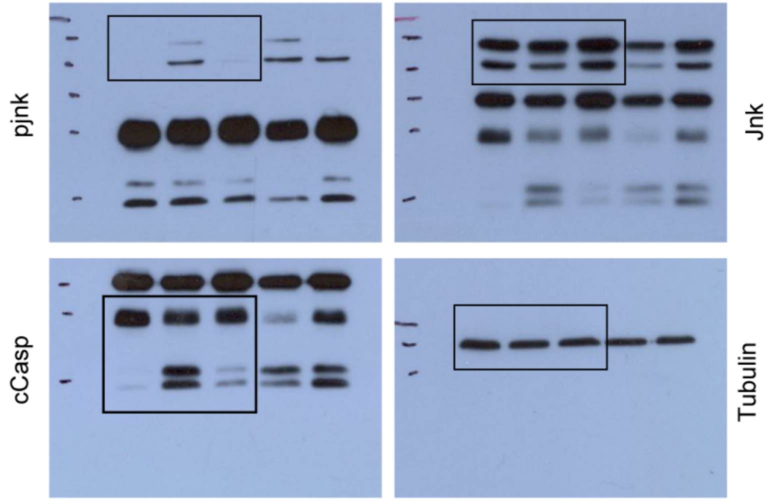

Supp2 G

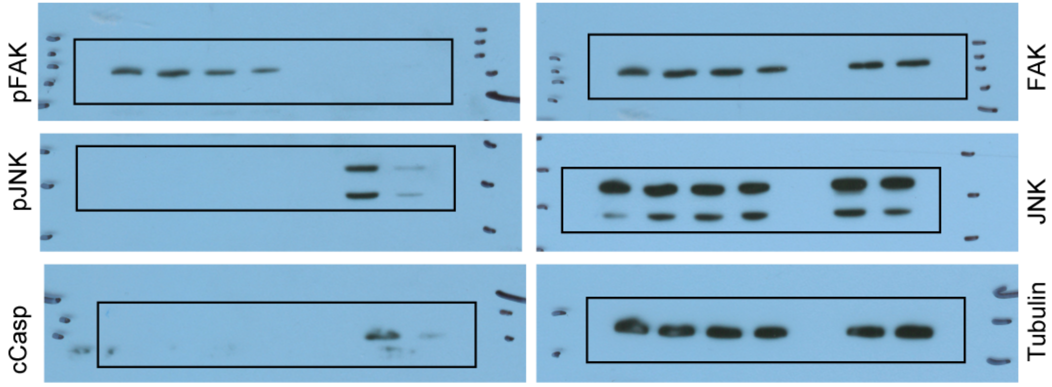

Figure 3 A

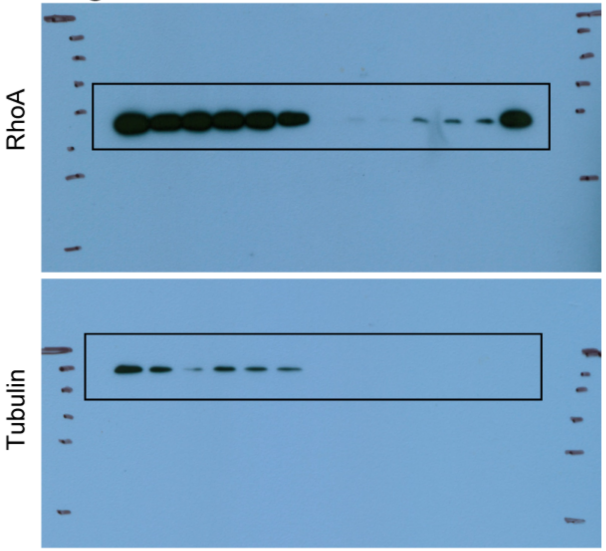

Figure 3 B

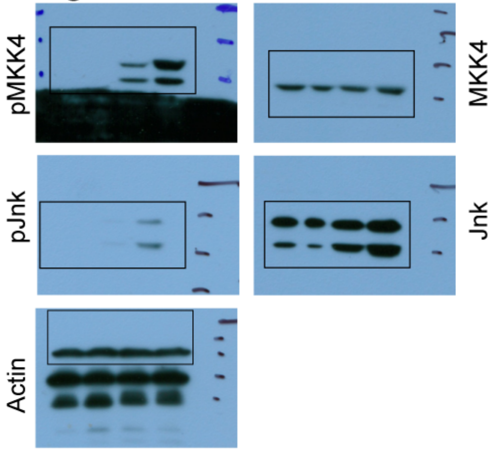

Figure 3 C

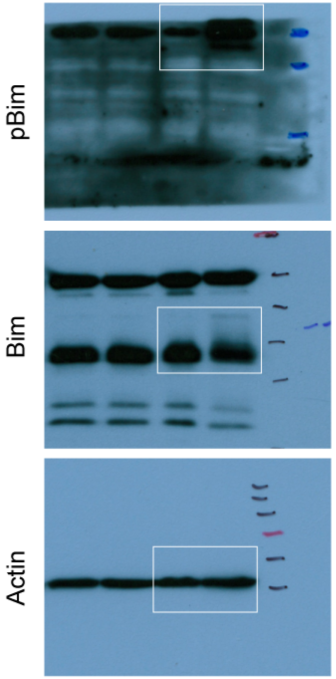

Figure 3 D

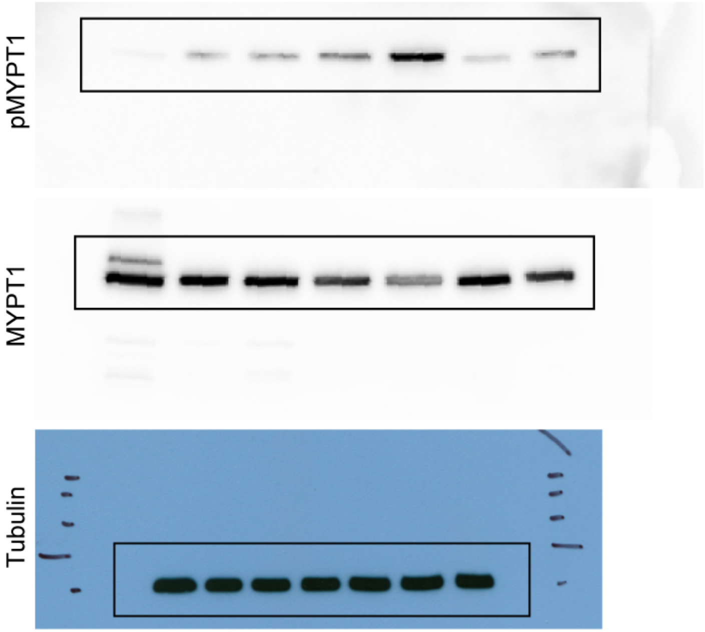

Supp 3 A

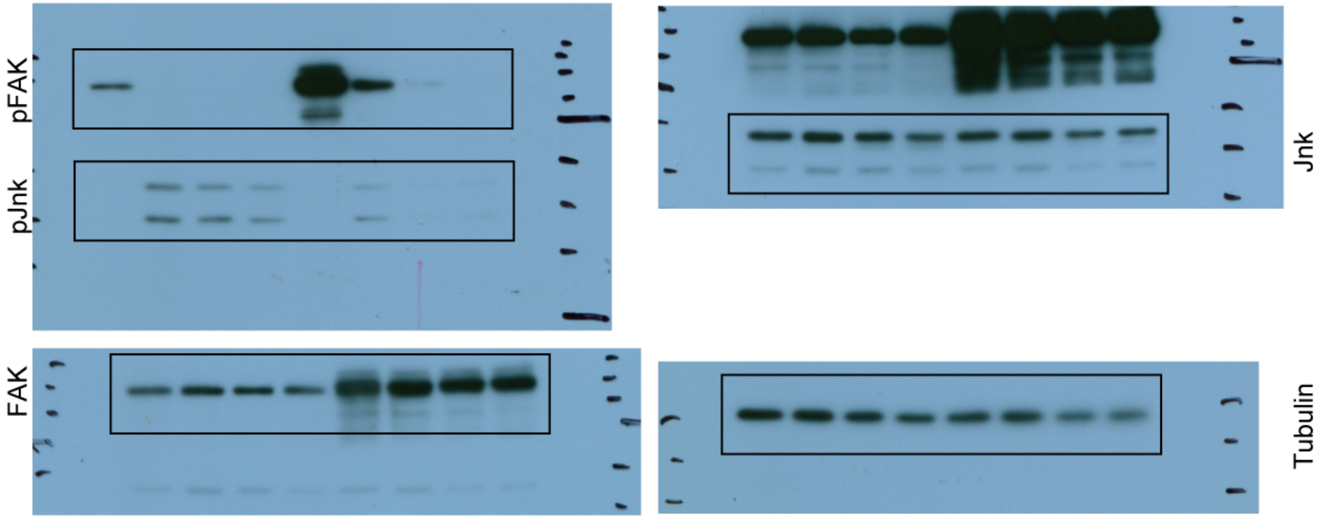

Supp 3 D

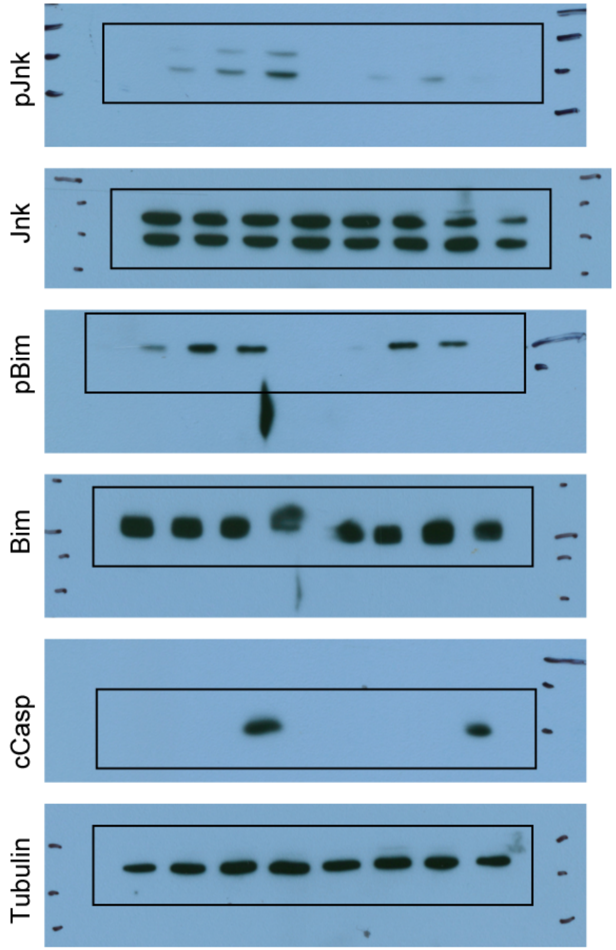

Supp 3 C

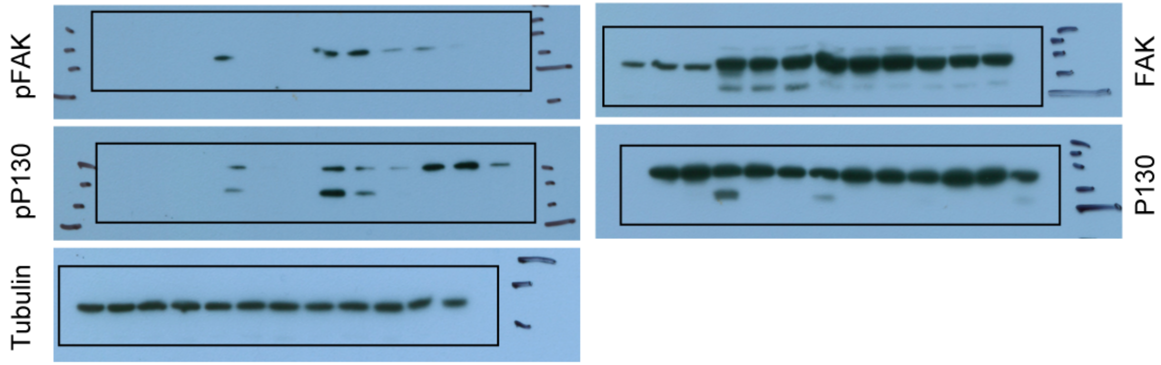

Figure 5 A

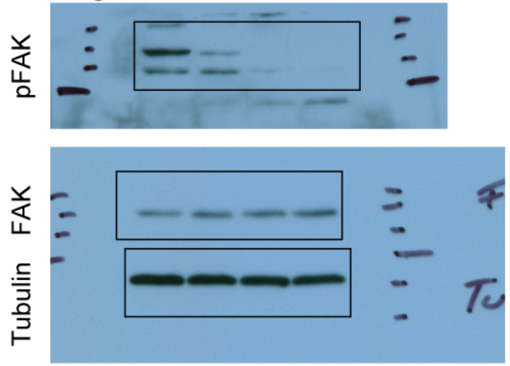

Figure 5 B

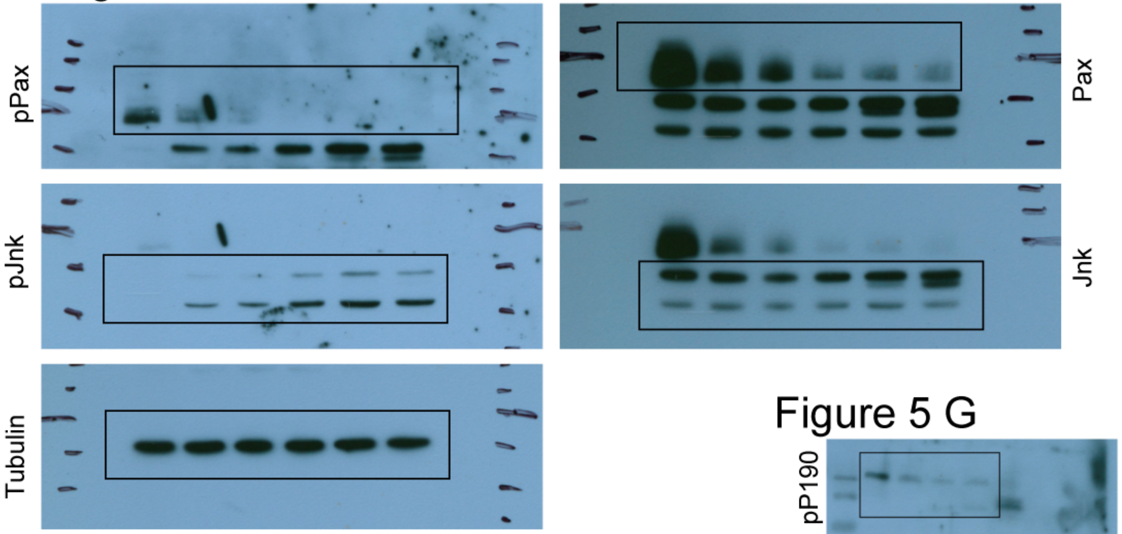

Figure 5 G

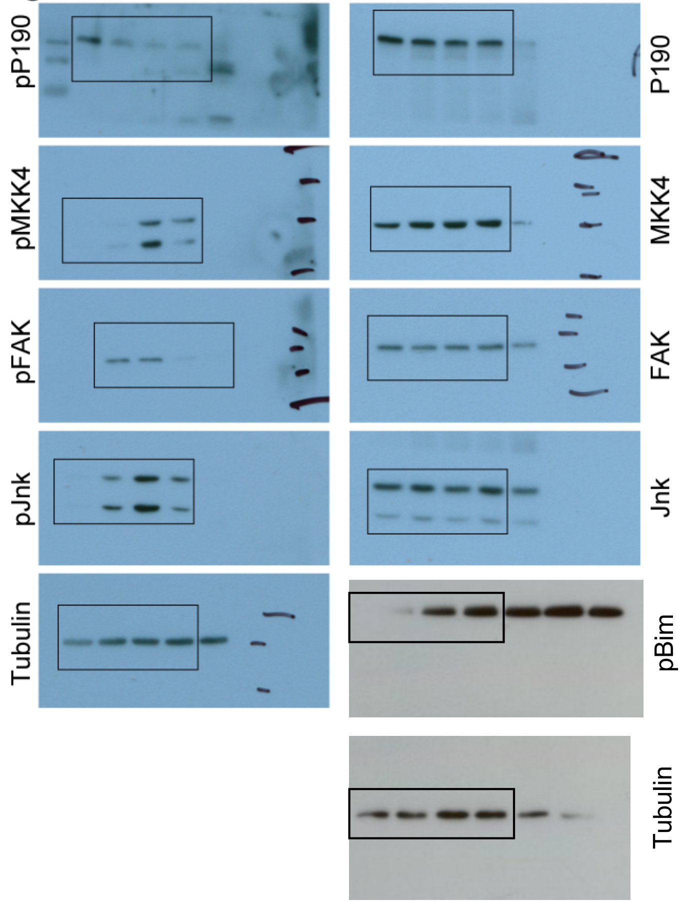

Figure 5 C

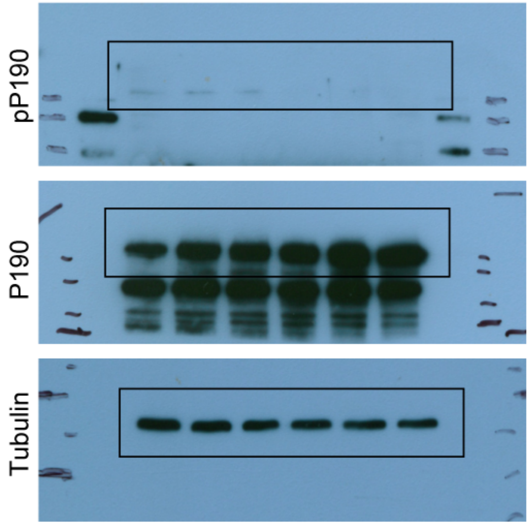

Figure 5 H

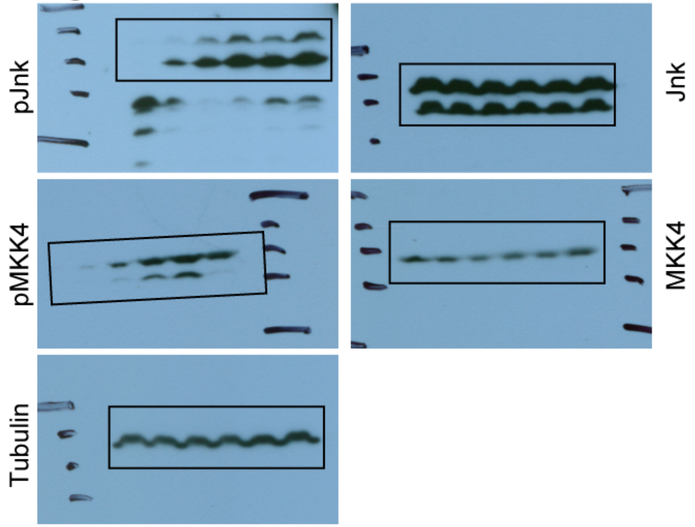

Supp 5 A

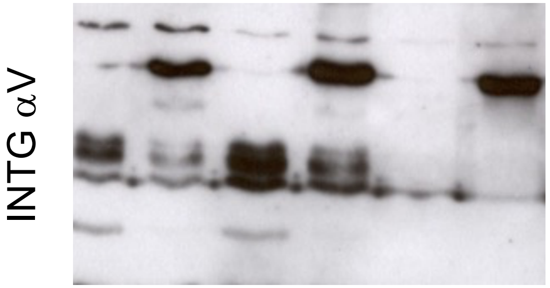

Supp 5 B

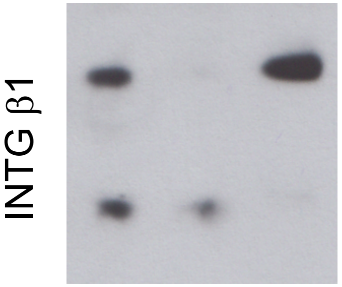

Supp 5 C

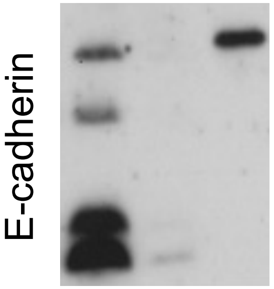

Supp 5 D

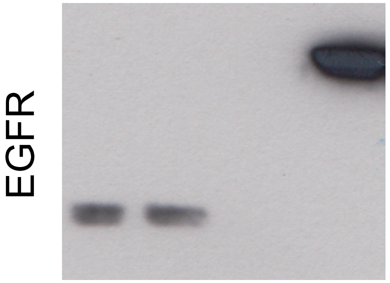

Supp 9 A left

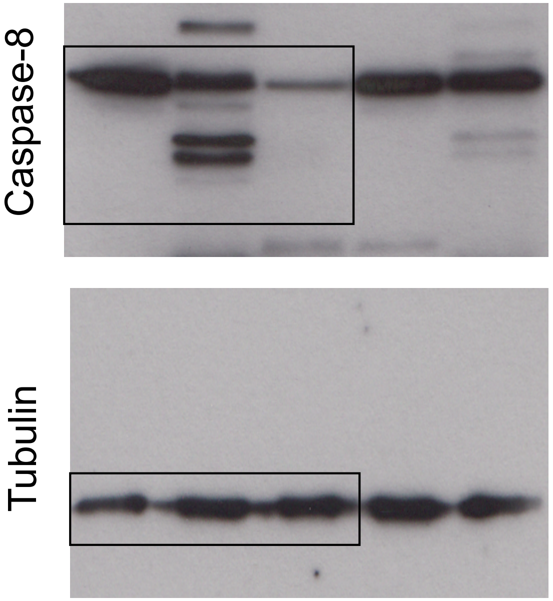

Supp 9 A right

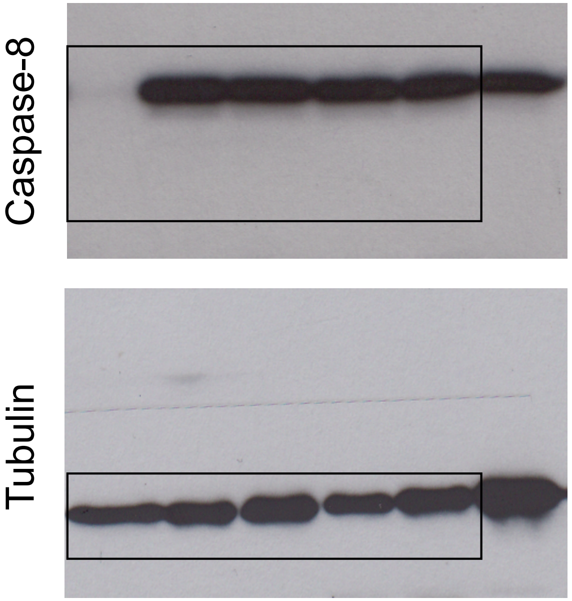

Figure 6 E

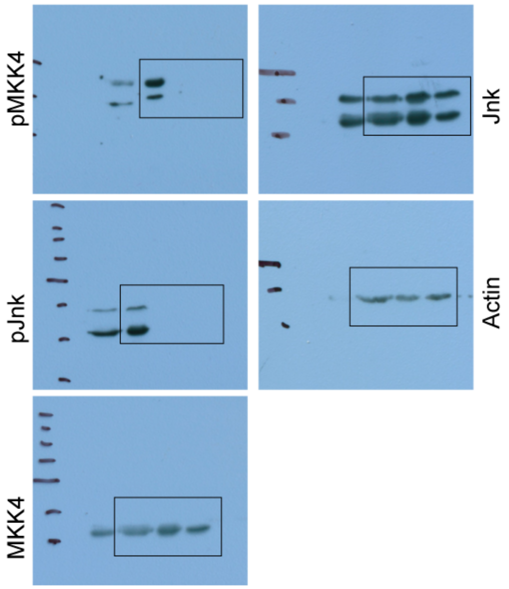

Supp 7 D_Top

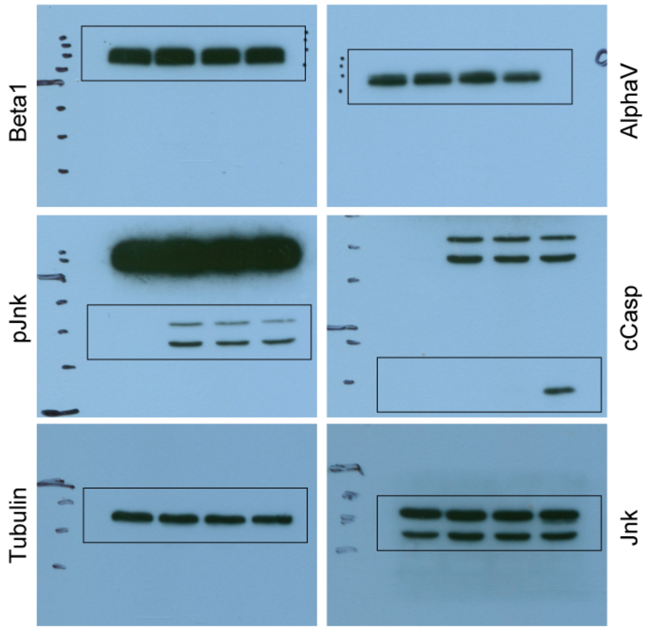

Supp 7 G

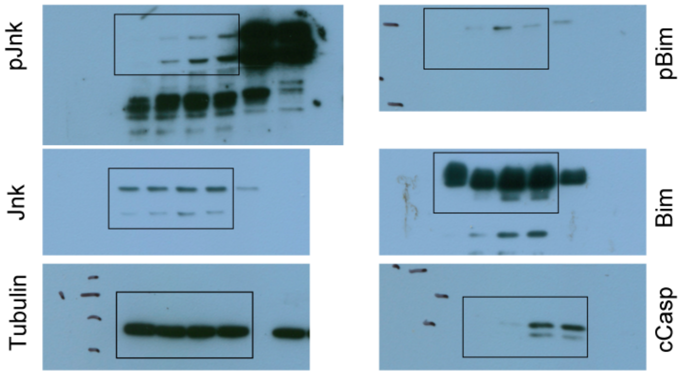

Supp 7 D_Bottom

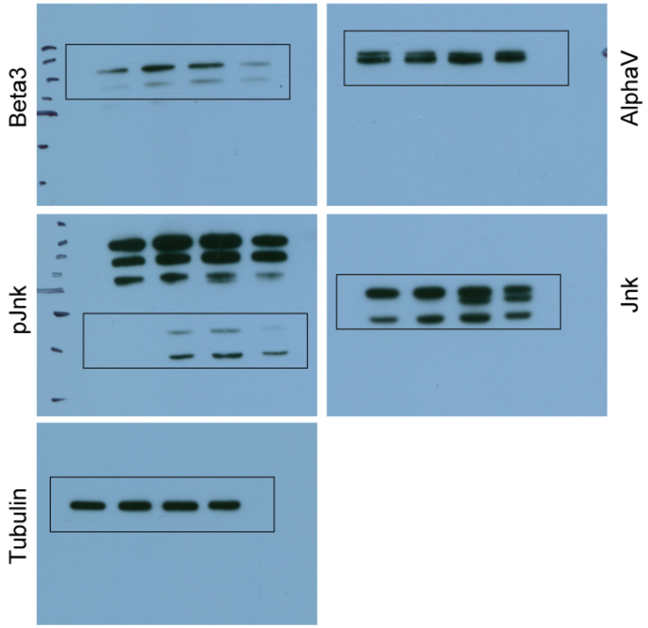

Supp 7 F

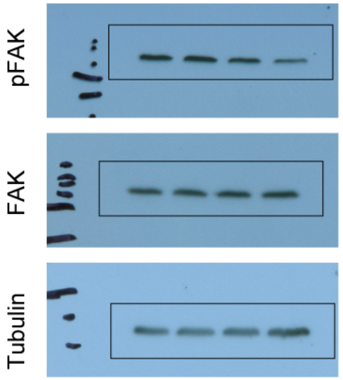

Figure 7 E

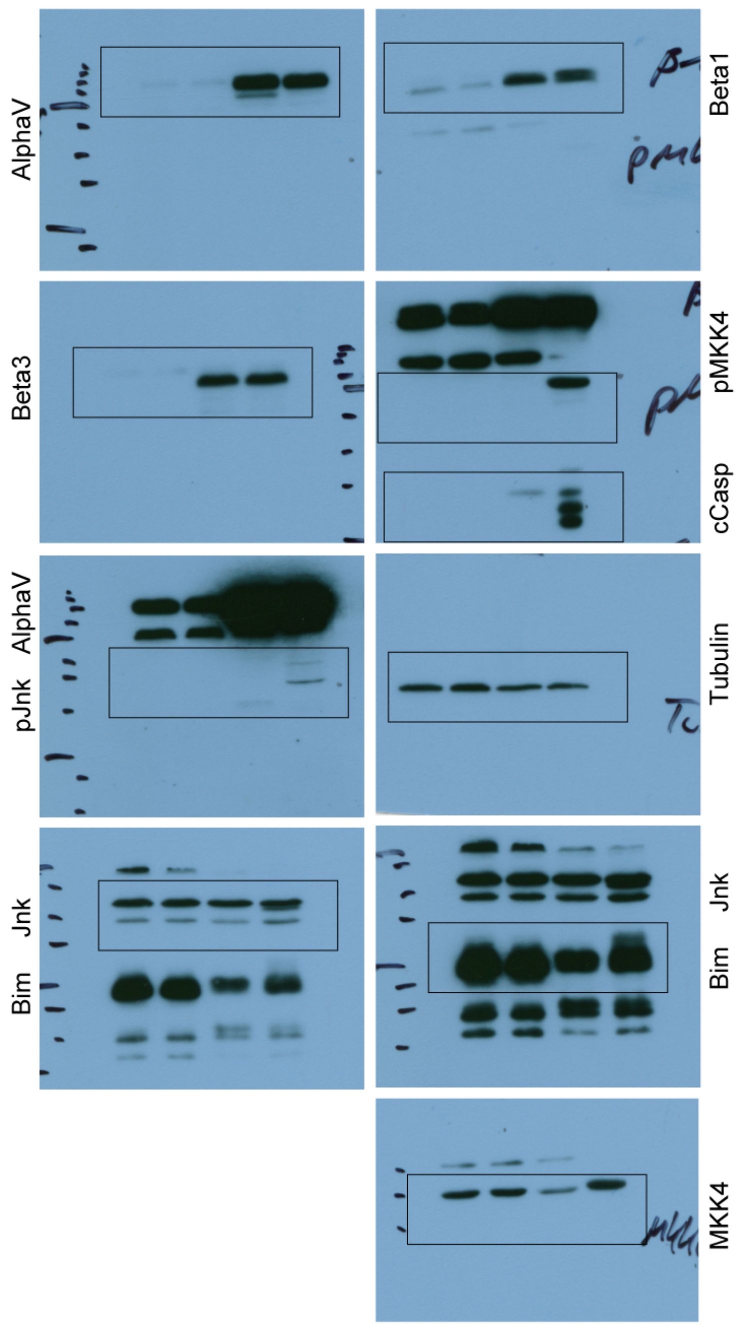

Supp 8 D

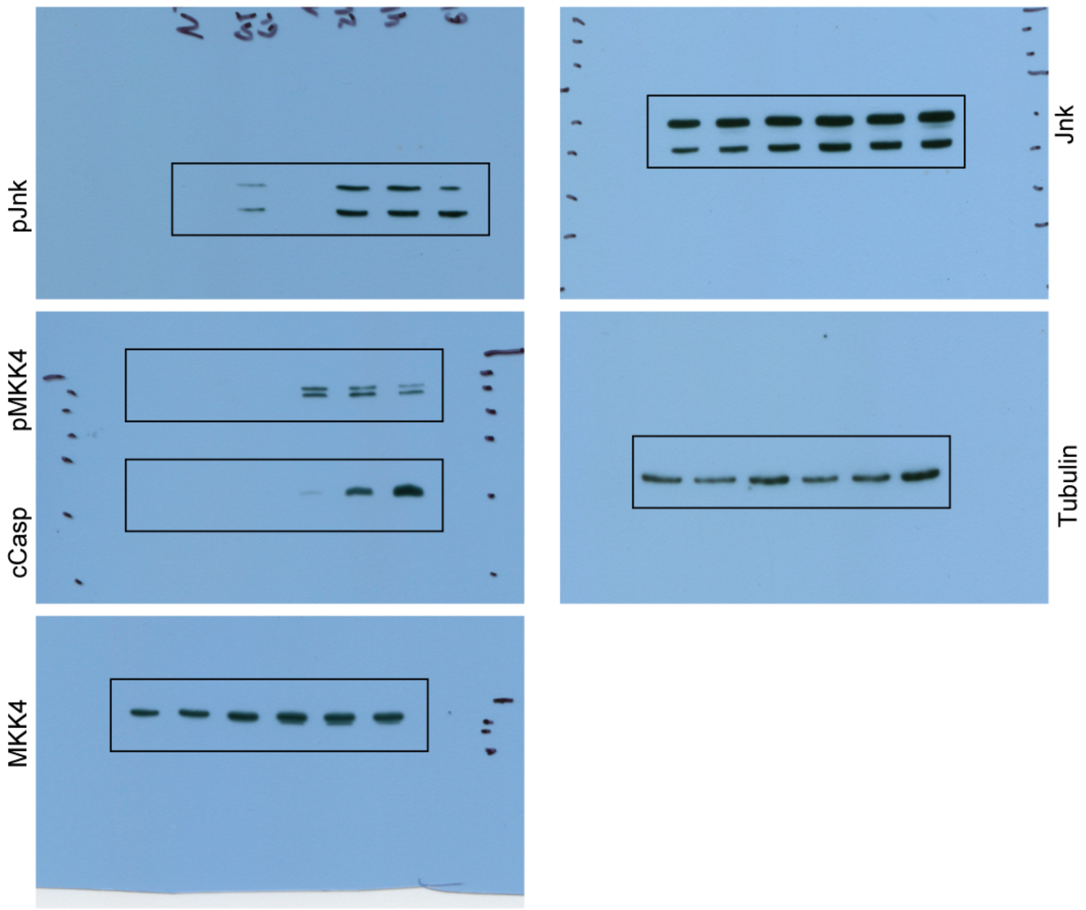

Supplementary Figure 10: Uncropped scans of all western blots
