# Supplementary material for: Identification of a novel anoikis signalling pathway using the fungal virulence factor gliotoxin
Source: Nat Commun. 2018 Aug 30;9:3524. doi: 10.1038/s41467-018-05850-w (PMC6117259; doi:10.1038/s41467-018-05850-w)
Supplement: Supplementary file 2 — Description of Additional Supplementary Files [file 41467_2018_5850_MOESM2_ESM.pdf]

Description of Additional Supplementary Files

File Name: Supplementary Movie 1
Description: GFP-paxillin is rapidly endocytosed followed by cell detachment. Confocal video time lapse microscopy showing the rapid translocation of stably transfected GFP-paxillin from focal adhesions at the plasma membrane to endocytic vesicles in response to 1 μM GT. This is followed by cell rounding and detachment after 14-18 min.
